# Crystalline silica particles cause rapid NLRP3-dependent mitochondrial depolarization and DNA damage in airway epithelial cells

**DOI:** 10.1186/s12989-020-00370-2

**Published:** 2020-08-10

**Authors:** Rongrong Wu, Johan Högberg, Mikael Adner, Patricia Ramos-Ramírez, Ulla Stenius, Huiyuan Zheng

**Affiliations:** Institute of Environmental Medicine, Karolinska Institutet, Box 210, SE-17177 Stockholm, Sweden

**Keywords:** Respirable crystalline silica particles, NLRP3 inflammasome, S198 pNLRP3, Mitochondrial depolarization, Carbonyl cyanide-*4*-(trifluoromethoxy) phenylhydrazone (FCCP), Double strand brakes (DSB), Non-homologous end joining (NHEJ), CC10, GPRC5A, Autotaxin (ATX)

## Abstract

**Background:**

Respirable crystalline silica causes lung carcinomas and many thousand future cancer cases are expected in e.g. Europe. Critical questions are how silica causes genotoxicity in the respiratory epithelium and if new cases can be avoided by lowered permissible exposure levels. In this study we investigate early DNA damaging effects of low doses of silica particles in respiratory epithelial cells in vitro and in vivo in an effort to understand low-dose carcinogenic effects of silica particles.

**Results:**

We find DNA damage accumulation already after 5–10 min exposure to low doses (5 μg/cm^2^) of silica particles (Min-U-Sil 5) in vitro. DNA damage was documented as increased levels of γH2AX, pCHK2, by Comet assay, AIM2 induction, and by increased DNA repair (non-homologous end joining) signaling. The DNA damage response (DDR) was not related to increased ROS levels, but to a NLRP3-dependent mitochondrial depolarization. Particles in contact with the plasma membrane elicited a Ser198 phosphorylation of NLRP3, co-localization of NLRP3 to mitochondria and depolarization. FCCP, a mitochondrial uncoupler, as well as overexpressed NLRP3 mimicked the silica-induced depolarization and the DNA damage response. A single inhalation of 25 μg silica particles gave a similar rapid DDR in mouse lung. Biomarkers (CC10 and GPRC5A) indicated an involvement of respiratory epithelial cells.

**Conclusions:**

Our findings demonstrate a novel mode of action (MOA) for silica-induced DNA damage and mutagenic double strand breaks in airway epithelial cells. This MOA seems independent of particle uptake and of an involvement of macrophages. Our study might help defining models for estimating exposure levels without DNA damaging effects.

## Introduction

The NLRP3 inflammasome is intensely studied and controls maturation of cytokines and innate inflammatory responses [1]. A wide variety of stimuli activate NLRP3 including microbes, endogenous danger signals (extracellular ATP, monosodium urate crystals (MSU)), or environmental particles (crystalline silica and asbestos). Activation includes assembly of NLRP3 monomers, ASC (apoptosis-associated speck-like protein), pro-caspase1, caspase1 activation, and secretion of IL-1β and IL-18 [1, 2]. NLRP3 activation is mainly studied in LPS-primed macrophages, and K^+^ efflux and NEK7 are critical for many activating stimuli [3], whereas phosphorylation of NLRP3 at serine 198 was recently found to be important for priming [4]. In epithelial cells the need for priming is not obvious [5–7].

Mitochondrial ROS (mtROS) induced by ATP, MSU crystals, silica and asbestos particles cause NLRP3 activation, often shown as IL-1β maturation [8, 9]. NLRP3 also senses mitochondrial dysfunction [10], and additional activating roles for mitochondria have been indicated. Thus, optimal NLRP3 activation by e.g. nigericin, but not by crystalline compounds such as Alum or MSU, depends on a translocation to mitochondria and the adaptor protein MAVS [11]. Mitochondria may also play a role in e.g. silica-activated NLRP3 via cardiolipin acting as a platform for NLRP3 [12]. Furthermore, cardiolipin participates in the LPS-induced and ROS-dependent priming in macrophages [2]. However, a recent study shows that several NLRP3 stimuli disperse the *trans*-Golgi network (TGN), and that dispersed TGN binds and activates NLRP3 in reconstituted HeLa cells and in macrophages. NLRP3-GFP did not co-localize with mitochondria after nigericin stimulation [13].

Respirable crystalline silica is carcinogenic and many thousand future lung cancer cases are expected in e.g. Europe [14]. A better understanding of mechanisms behind DNA damage and mutations in the respiratory epithelium [15, 16], should possibly underpin a lowered permissible exposure level and thus also a reduced cancer risk. We have used lung epithelial cell models for genotoxicity studies [6]. Employing these models, we previously showed that low doses of silica particles induced NLRP3 activation and double strand breaks (DSBs) in a process involving an ATM-dependent secretion of autotaxin (ATX) [6]. ATX is of possible importance for epithelial paracrine defensive or pathological adaptations [17].

Here we present novel in vitro and in vivo evidence for rapid DNA damaging effects of silica in epithelial cells. We find that silica particles in low doses and within 3–10 min induce serine 198 NLRP3 phosphorylation, mitochondrial depolarization, and DNA damage accumulation. This scenario distinguishes the rapid genotoxicity documented here from earlier described genotoxic responses.

## Results

### Silica-induced NLRP3 activation and mitochondrial depolarization

We investigated short-term effects of silica exposure and used human immortalized lung bronchial epithelial cells 16HBE14o- (16HBE). The used particles were characterized previously [6], and the selected dose (5 μg/cm^2^) was derived from our earlier study, which showed DNA damage responses at doses of 0.1–10 μg/cm^2^ 16 h after silica addition [6]. 5 μg/cm^2^ was designated as a LOEL for in vitro genotoxicity [16].

**Fig. 1:**
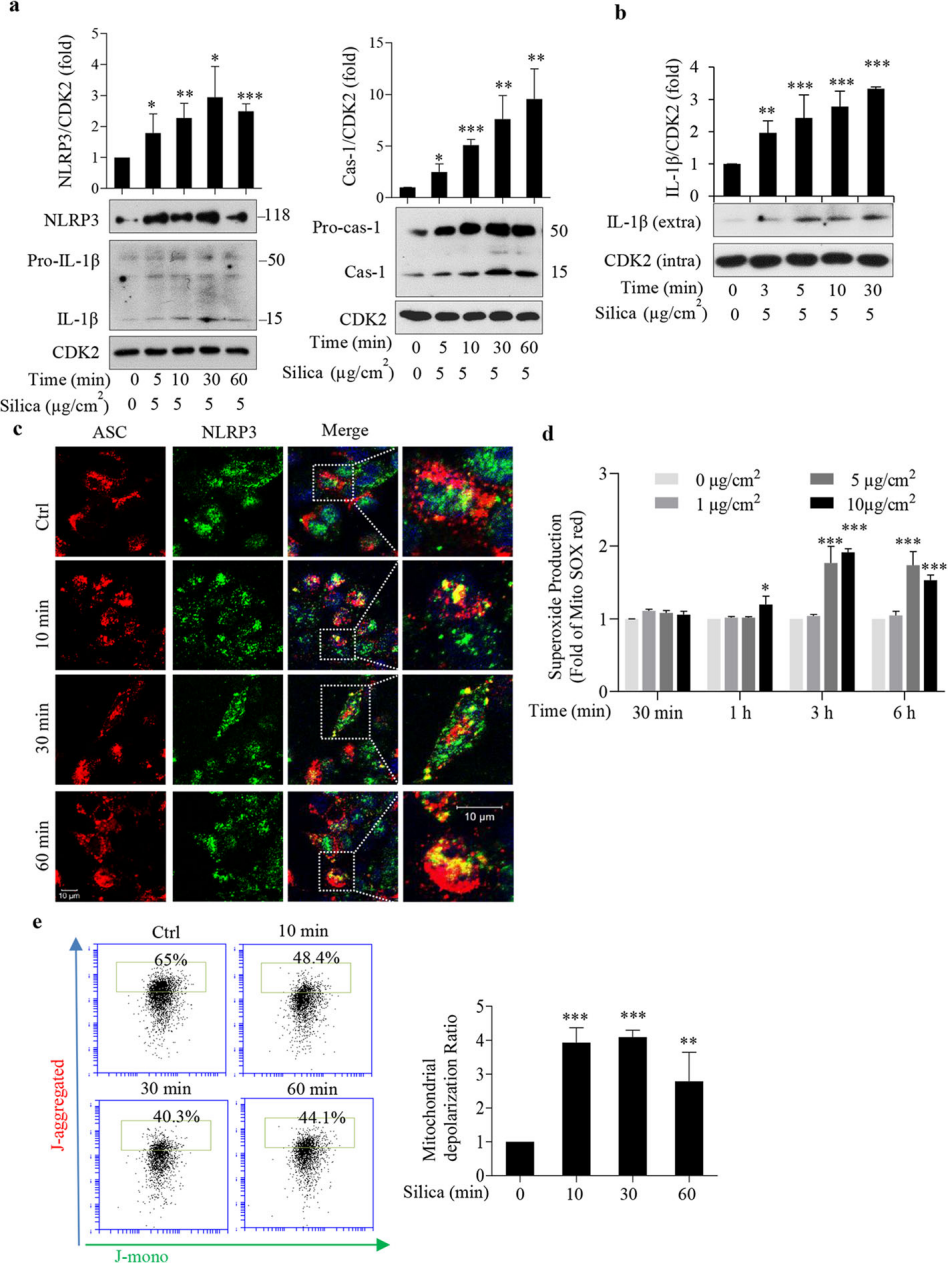
Silica induces rapid NLRP3 activation without increased mtROS production. **a, b** Western blot analysis of NLRP3, caspase-1, IL-1β of cell lysate (**a**) or supernatant (**b**) from 16HBE cells treated with silica (5 μg/cm^2^) for times indicated. **c** Representative confocal images showing co-localization of NLRP3 (green) and ASC (red) in 16HBE cells treated with silica. **d** Mitochondrial ROS in silica-exposed 16HBE cells. Concentrations and times indicated. **e** Mitochondrial membrane potential measurements using JC-1 staining in silica-treated (5 μg/cm^2^) 16HBE cells. The ratio of green to red fluorescence was used for assessing the mitochondrial membrane potential and depolarization ratio. Bars show means ± SD. All experiments were performed at least in triplicate. *p < 0.05, **p < 0.01, ***p < 0.001 versus control as determined by ANOVA

As shown in Fig. 1a,b, NLRP3 and caspase-1 were up-regulated already at 10 min. Cleavage of IL-1β was seen also at 10 min (Fig. 1b), and earlier than previously reported in epithelial cells [5–7]. Confocal microscopy showed co-localization of NLRP3 and its adaptor protein ASC at 10 min, persisting for at least 60 min (Fig. 1c). These data indicate that silica induces NLRP3 inflammasome activation within 10 min. A similar, perhaps related, short time span has been shown for effects on Ca^2+^ levels and for the inhibition of the cation channel TRPV4 induced by silica nanoparticles in 16HBE cells [18].

We also measured mitochondrial ROS (mtROS) levels after silica addition. Figure 1d shows that mtROS was significantly increased at 3 h and 6 h. In line with previous observations on a basal ROS level [19], we detected a steady-state ROS level during the first 30 min. The antioxidant MitoTEMPO prevented the increase seen at 3 and 6 h, confirming a mitochondrial origin (Additional file 1: Fig. S1b). We saw no effect on total ROS generation until 3 h after silica addition (Additional file 1: Fig. S1a). However, we found that silica decreased the mitochondrial membrane potential after 10 min exposure. Silica shifted the mitochondria population from J-aggregated to J-mono (Fig. 1e). This effect is also displayed as a 4-fold increase in depolarization (Fig. 1 e). The effect of silica on membrane potential was also evaluated with TMRE (Additional file 1: Fig. S3a), which confirmed a depolarization at 10 min.

### Mitochondrial membrane uncoupler, FCCP, induces similar effects as silica particles

To explore the mitochondrial effects further, we tested the mitochondrial inhibitors antimycin A and FCCP. Antimycin A inhibits complex III of the mitochondrial respiratory chain and results in mtROS generation [20]. Fig. S2a (Additional file 1) confirms that low concentrations (1 μM) of Antimycin A significantly increased mtROS at 30 min and later. Higher concentrations (50 μM) also induced depolarization (Additional file 1: Fig. S2b).

**Fig. 2:**
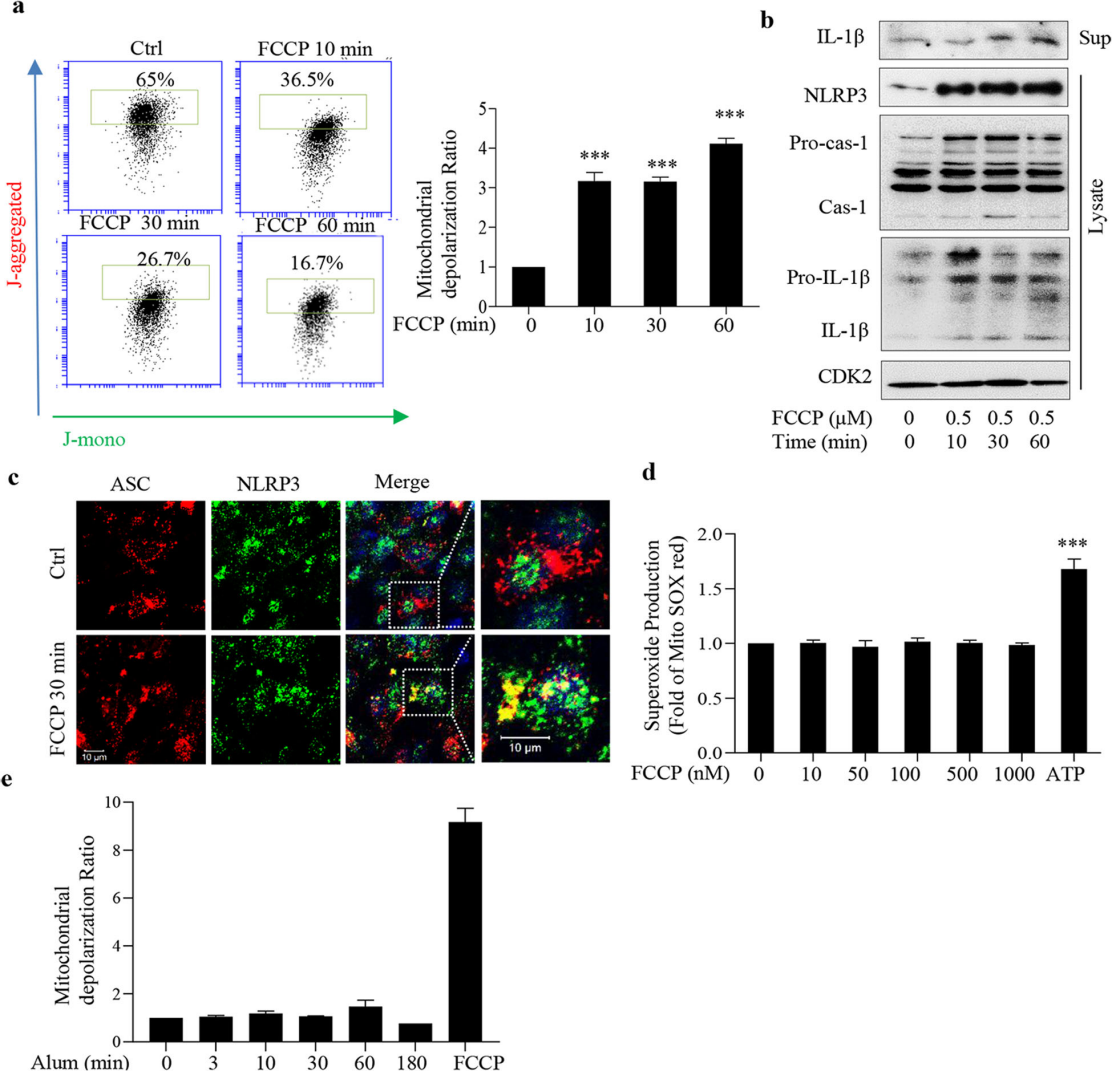
Low concentrations of FCCP induce NLRP3 activation. **a** Mitochondrial membrane potential measurement using JC-1 staining in 16HBE cells treated with FCCP (500 nM) at indicated time points. **b** Western blot analysis of NLRP3, caspase-1, IL-1β of cell lysate or supernatants from 16HBE cells treated with FCCP (500 nM) for times indicated. **c** Representative confocal images showing co-localization of NLRP3 (green) and ASC (red) in 16HBE cells treated with FCCP (500 nM) for 30 min. **d** Mitochondrial ROS measurement in 16HBE cells treated with indicated concentrations of FCCP for 30 min or with ATP (15 min, 1 mM). **e** Mitochondrial membrane potential measurement using JC-1 staining in 16HBE cells treated with aluminum particles (25 μg/ml) for indicated times, or with FCCP (10 min, 10 μM). Bars show means ± SD. All experiments were performed at least in triplicate. ***p < 0.001 compared to untreated cells as determined by ANOVA

FCCP is an uncoupler of the electron transport chain inducing oxidative phosphorylation, which decreases the mitochondrial membrane potential, and also the plasma membrane potential [21]. We found that 500 nM FCCP induced a similar mitochondrial depolarization as silica (Fig. 2a). Using TMRE as a probe, we confirmed an effect at 10 min (Additional file 1: Fig. S3a). Unexpectedly, FCCP exposure also cleaved caspase1 and processed IL-1β within 10 min (Fig. 2b). We also found that FCCP (500 nM) induced a co-localization of NLRP3 and ASC, as indicated by confocal microscopy (Fig. 2c). Furthermore, 500 nM FCCP (30 min up to 3 h) did not increase mtROS levels (Fig. 2d), although high doses of FCCP-induced loss of mitochondrial membrane potential may increase mtROS [10]. Thus, 500 nM FCCP induced a similar NLRP3 inflammasome activation and mitochondrial depolarization as did silica (Fig. 1e). In additional studies we used aluminum particles. As indicated in Fig. 2e, we observed no depolarization in response to these particles.

We analyzed the medium for cell leakage. Fig. S3b and c (Additional file 1) shows that neither silica nor FCCP in used concentrations increased LDH release under the studied conditions. This indicates a lack of cell damage and this was expected as higher concentrations (10 μg/cm^2^) did not affect LDH levels [6]. We also tested for early signs of apoptosis by employing Annexin V and PI staining. We found no indications of increases for up to 24 h, whereas the positive control gave a clear response (Additional file 1: Fig. S3d,).

### NLRP3 is essential for rapid DNA damage response (DDR)

**Fig. 3:**
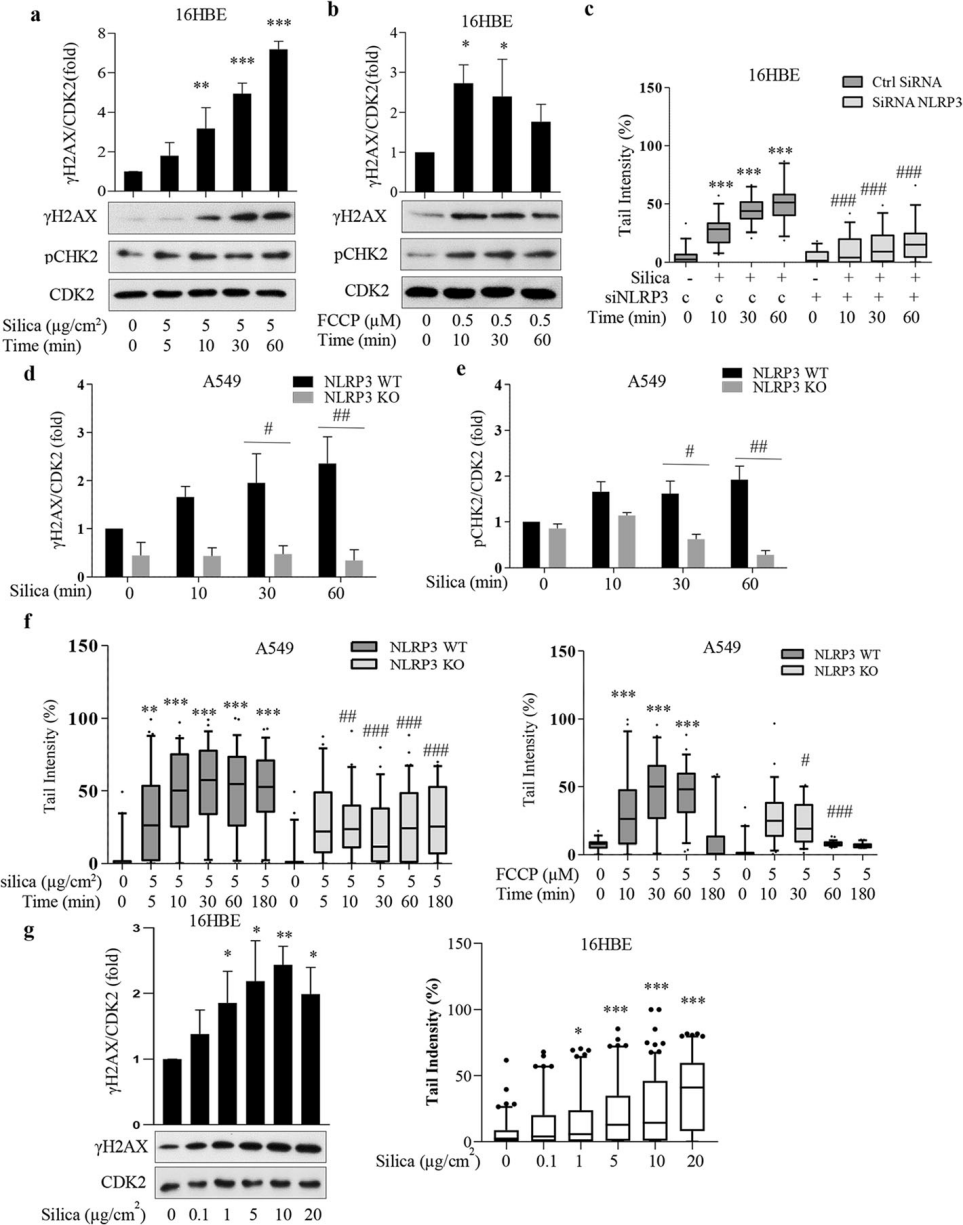
Silica and FCCP rapidly induce DNA damage. **a, b** Western blot analysis for pCHK2 and γH2AX in cell lysate from 16HBE cells treated with silica (5 μg/cm^2^) or FCCP (500 nM) at times indicated. **c** Comet assay analysis of DNA damage in 16HBE cells transfected with siRNA NLRP3 (+) or control siRNA (**c**) for 72 h and thereafter exposed to silica (5 μg/cm^2^) for times indicated. **d, e** Western blot analysis of γH2AX (**d**) and pCHK2 (**e**) in cell lysate from A549 WT or A549 NLRP3 knock out (CRISPR-CAS9) cells treated with silica (5 μg/cm^2^) for times indicated. **f** Comet assay analysis of DNA damage in A549 WT or A549 NLRP3 knock out cells treated with silica (5 μg/cm^2^) or FCCP (5 μM). **g** Western blot analysis for γH2AX (left) and Comet assay analysis of DNA damage (right) in 16HBE cells treated with silica (from 0.1 to 20 μg/cm^2^) for 10 min. Bars in **a**, **b**, **d** and **e** show means ± SD. Boxplots in **c** and **f** show median with 95% confidence interval. All experiments were performed at least in triplicate. *p < 0.05, **P < 0.01, ***P < 0.001 compared to untreated cells, or #*p* < 0.05, ##*p* < 0.01, ###*p* < 0.001 compared to non-transfected cells, as determined by ANOVA

Next we analyzed the induction of nuclear DNA damage in 16HBE cells. Figure 3a and b show that not only silica but also FCCP caused activation of the DNA damage markers, γH2AX and pCHK2, within 10 min. Comet assay, which detects DNA strand breaks, confirmed that DNA damage was induced (Fig. 3c). We studied the dose-response for silica and found that 1 μg/cm^2^ and more induced a significant γH2AX and Comet assay responses as detected at 10 min (Fig. 3g). In line with a previous study documenting a DDR of silica after 8 h [22], we found that siRNA NLRP3 attenuated the DDR (Fig. 3c, Additional file 1: Fig. S4 a, b). We also confirmed a rapid (10 min) silica-induced phosphorylation of ATM (pATM) [6]. siRNA NLRP3 inhibited this effect (Additional file 1: Fig. S4a). Furthermore, we analyzed γH2AX levels in experiments with Antimycin A (Additional file 1: Fig. S2). As for silica, γH2AX levels did not correlate with mtROS levels, but rather with depolarization (Additional file 1: Fig. S2b and c). These data indicated deviations from previous studies [6, 15, 16] and spurred continued investigations.

**Fig. 4:**
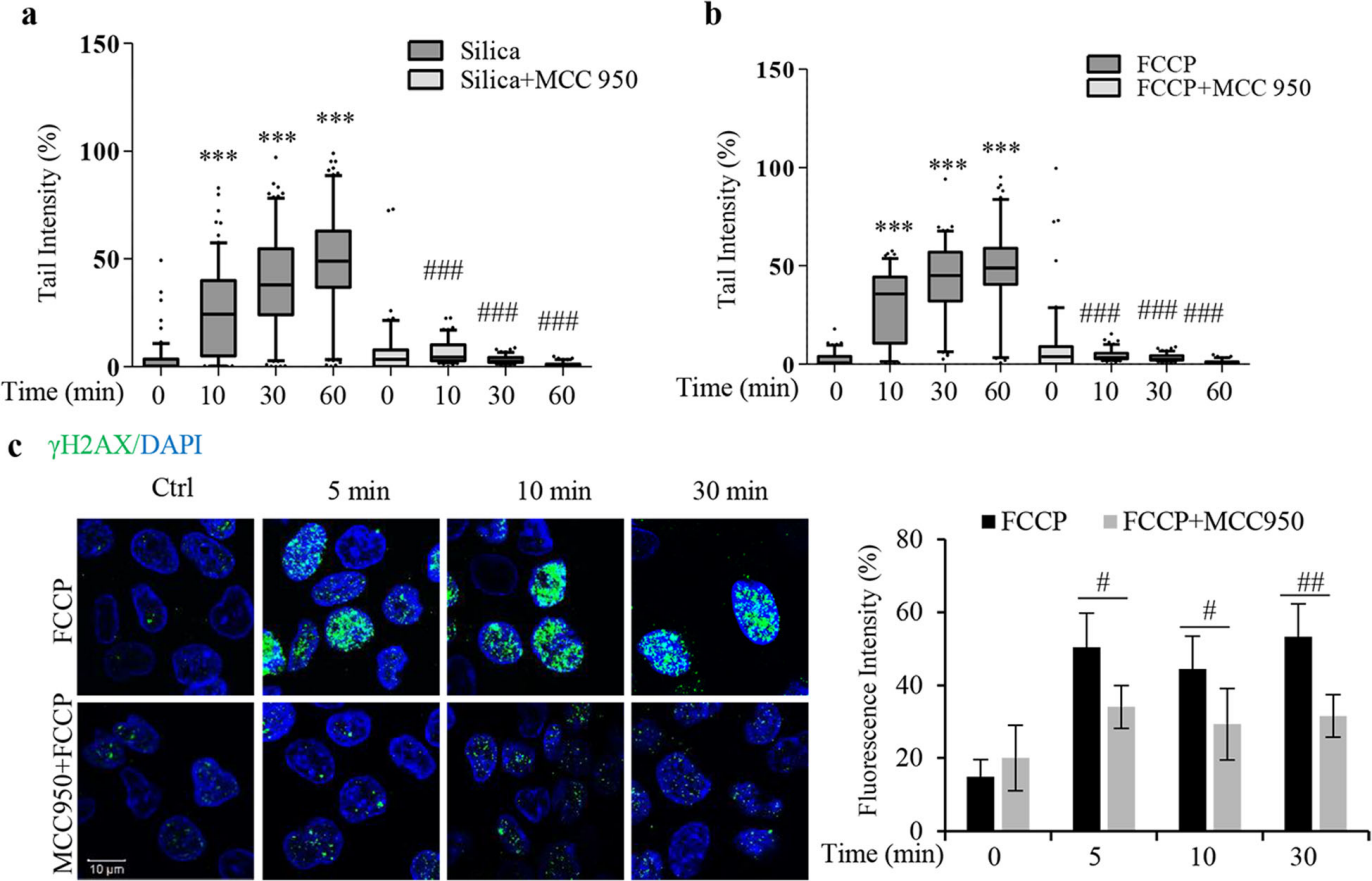
The NLRP3 inhibitor MCC950 inhibits silica- and FCCP-induced DNA damage. **a**, **b** Comet assay analysis of DNA damage in 16HBE cells pretreated with MCC950 (100 nM) for 1 h and thereafter with silica (5 μg/cm^2^) (**a**) or FCCP (500 nM) (**b**) for times indicated. **c** Representative confocal images showing γH2AX (green) staining in 16HBE cells pretreated (1 h) with MCC950 (100 nM) and thereafter treated with FCCP (500 nM) as indicated. Cell nuclei were stained with DAPI (blue). Bars show fluorescence intensity of γH2AX from 100 cells. Boxplots in **a** and **b** show median with 95% confidence interval. All experiments were performed at least in triplicate. ***p < 0.001 compared to untreated cells, or #*p* < 0.05, ##*p* < 0.01, ###*p* < 0.001 compared to cells not exposed to inhibitor, as determined by ANOVA

To get stable NLRP3 knockout (KO) cells we employed A549 cells (an alveolar Type II epithelial cell line) treated with a CRISPR CAS9 plasmid. Fig. S4c (Additional file 1) shows the downregulation efficiency. These cells had a lower background level of γH2AX (Fig. 4d). In line with the siRNA NLRP3 experiments using 16HBE cells, we saw attenuated γH2AX and pCHK2 responses in silica- or FCCP-treated A549 NLRP3 KO cells (Fig. 3d, e, Additional file 1: Fig. S4d). In addition, the Comet assay response to both silica and FCCP was decreased in NLRP3 KO cells (Fig. 3f). These data indicate that NLRP3 is essential for the early DDR induced by silica and FCCP.

We employed camptothecin, a drug that does not produce ROS [6]. We found that camptothecin induced a clear γH2AX response (Additional file 1: Fig. S4e). Thus, camptothecin, which acts as a topoisomerase inhibitor [6] gave a similar γH2AX response as silica. This effect was independent of NLRP3 as NLRP3 KO did not change the response (Additional file 1: Fig. S4e).

In additional experiments with 16HBE cells we tested MCC950, a small molecule that inhibits the NLRP3-ASC complex formation via unknown mechanisms [23, 24]. MCC950 reduced silica- and FCCP-induced DNA damage in 16HBE cells (Fig. 4a, b). Confocal microscopy confirmed the results shown in NLRP3 KO cells (Fig. 3d) and shows that MCC950 pretreatment reduced the FCCP-induced γH2AX nuclear foci (Fig. 4c). Taken together, these data indicate an essential role for NLRP3 in the rapid DNA damage response.

### Mitochondrial depolarization is essential for the silica-induced rapid DNA damage

**Fig. 5:**
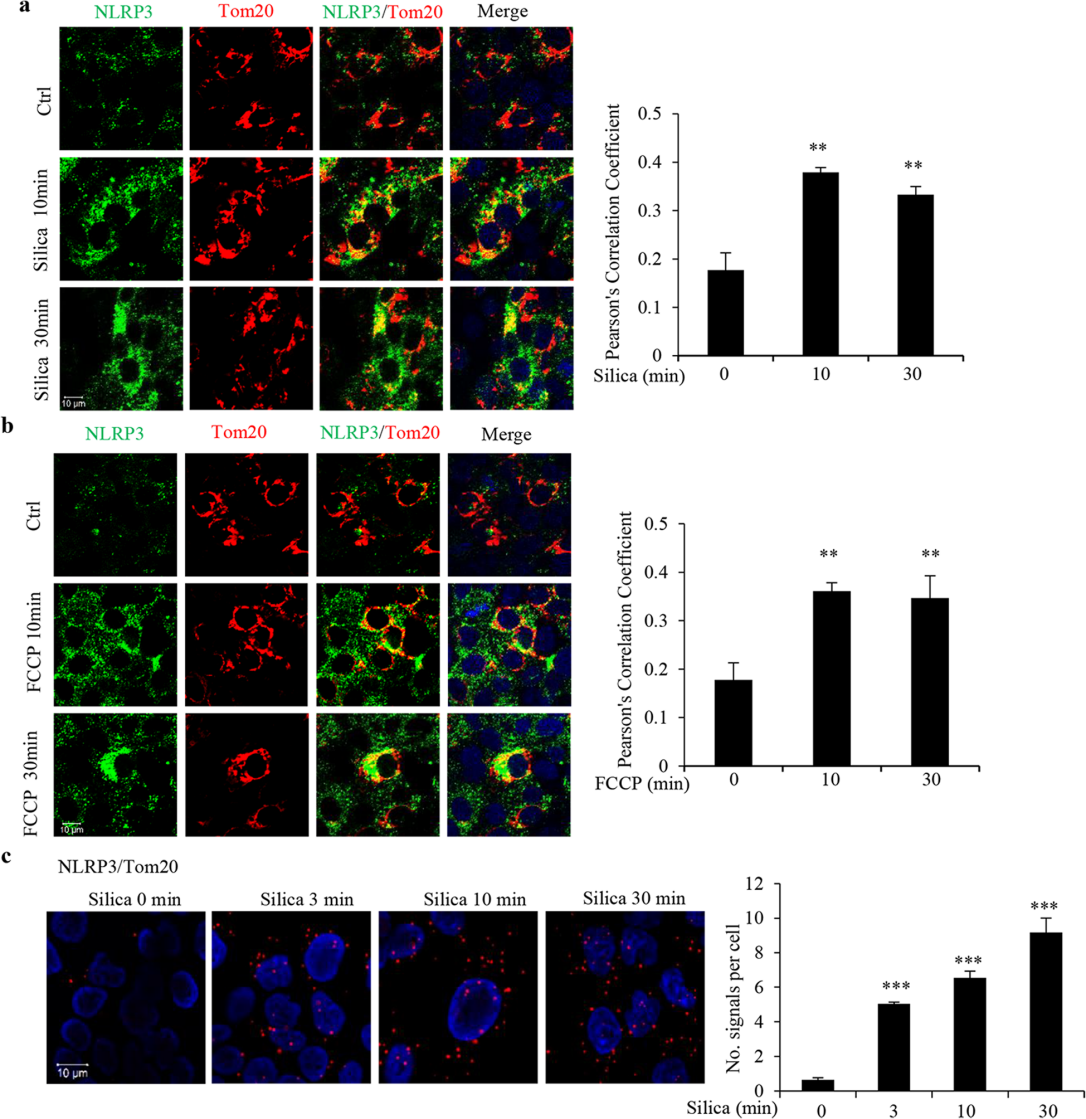
Silica and FCCP rapidly induce NLRP3 recruitment to mitochondria in 16HBE cells. **a, b** Representative confocal images showing co-localization of Tom20 (red) and NLRP3 (green) in cells treated with silica (5 μg/cm^2^) (**a**) or FCCP (500 nM) (**b**) for times indicated. Bars show the co-localization as calculated by Pearson’s correlation coefficient. Data from 100 cells were analyzed. **c** PLA assay analysis for co-localization of NLRP3 and Tom20 in cells treated with silica (5 μg/cm^2^) for times indicated. Red dots indicate proximity between NLRP3 and Tom20. Nuclei were stained with DAPI (blue). Bars show means ± SD. All experiments were performed at least in triplicate. **p < 0.01 compared to untreated cells as determined by ANOVA

Physical contact between NLRP3 and the mitochondrial outer membrane has been reported [2, 11, 12]. We thus examined NLRP3-mitochondria proximity in silica-treated 16HBE cells. Figure 5a shows that in control conditions there was no co-localization while silica (5 μg/cm^2^) induced a co-localization between NLRP3 and Tom20, a marker for the outer mitochondrial membrane. This was seen within 10 min. Co-localization was confirmed by employing PLA assay (Fig. 5c). FCCP (500 nM, 10 min and 30 min) also induced NLRP3 co-localization with Tom20 (Fig. 5b). These data indicate that silica- and FCCP-induced mitochondrial depolarization involves recruitment of NLRP3 to mitochondria. The co-localization is in line with some previous studies using non-particle stimulators, and documenting translocations, however at later time points [2, 11, 12].

**Fig. 6:**
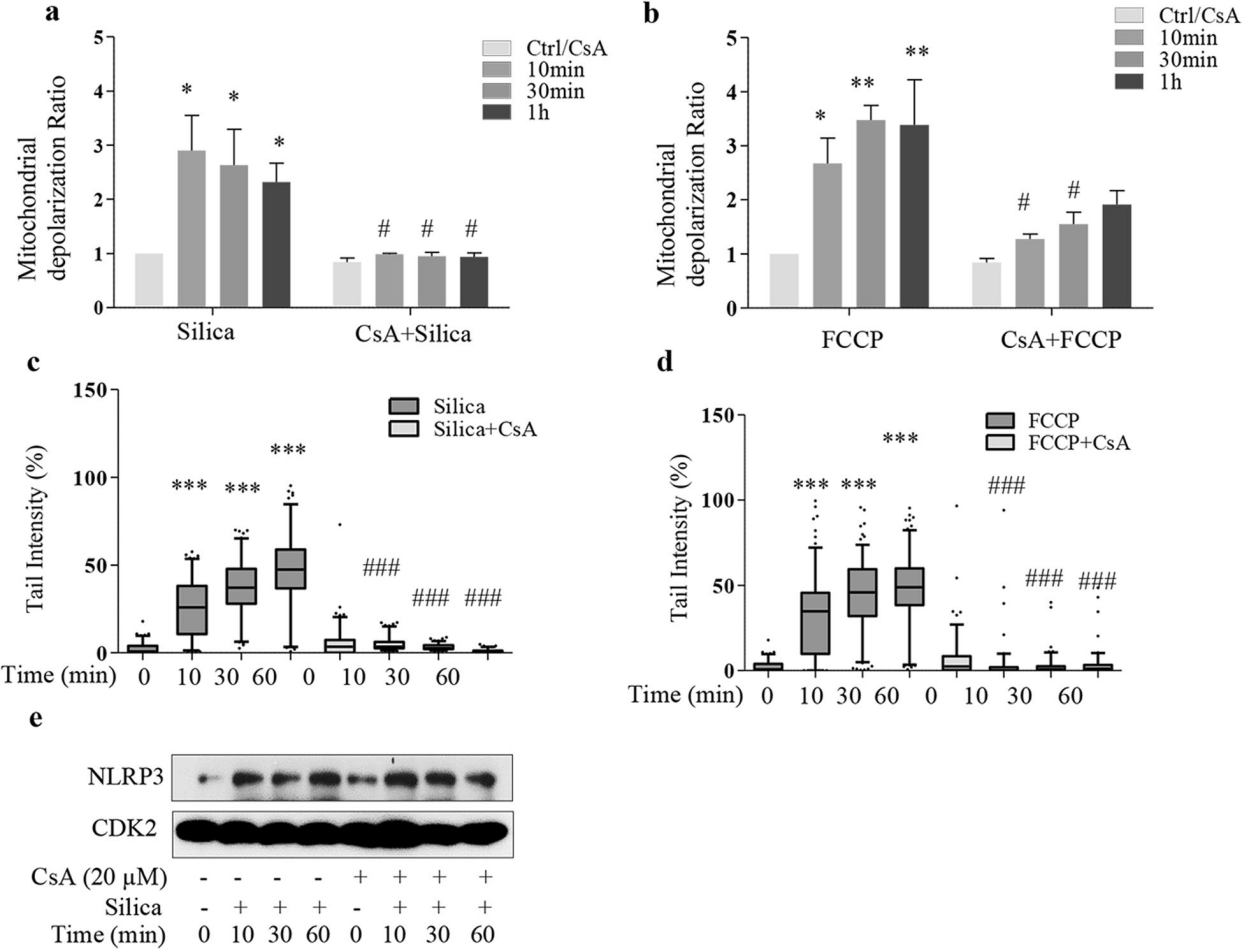
Cyclosporine A (CsA) stabilizes the membrane potential and prevents DNA damage. **a, b** Mitochondrial membrane potential (using JC-1 staining) in 16HBE cells pretreated with CsA (10 μM) for 1 h and thereafter with silica (5 μg/cm^2^) (**a**) or FCCP (500 nM) (**b)** for times indicated. The ratio of green to red fluorescence was used for assessing the mitochondrial membrane potential and depolarization ratio. **c, d** Comet assay analysis of DNA damage in cells from **a** and **b**. Bars in **a** and **b** show means ± SD. **e** Western blot analysis of NLRP3 in 16HBE cells pretreated with CsA (10 μM) for 1 h and thereafter with silica (5 μg/cm^2^). All experiments were performed at least in triplicate. Boxplots in **c** and **d** show median with 95% confidence interval. **e** Western blot analysis of NLRP3 of cell lysate from 16HBE cells pretreated with CsA. **a**. *p < 0.05, **p < 0.01, ***p < 0.001 compared to untreated cells, or #*p* < 0.05, ###*p* < 0.001 compared to cells not exposed to inhibitor, as determined by ANOVA

Next, we employed cyclosporine A (CsA), which inhibits the mitochondrial membrane permeability transition pore and stabilizes mitochondria [25]. Figure 6a and b show that CsA prevented silica-induced membrane depolarization in 16HBE cells. Comet assay shows that CsA also reduced both the silica- and FCCP-induced DNA damage responses (Fig. 6c, d). The NLRP3 response, however, was not affected (Fig. 6e). The CsA data confirm a role for mitochondria and suggest that a NLRP3-dependent mitochondrial depolarization is essential for silica-induced DNA damage.

### Silica - cell membrane contact induces NLRP3 S198 phosphorylation and mitochondrial depolarization

Histology shows intra-cellular silica particles in respiratory epithelium from silicotic patients [26], and we investigated the time-course for particle uptake in vitro, employing transmission electron microscopy (TEM). We found that already at 5 min of exposure many silica particles were in contact with the cell membrane, and they affected its outline. At 10 min we observed long cell protrusions exposing large membrane areas to the particle surfaces (Additional file 1: Fig. S5a) and resembling previously described ruffling [27] and phagocytic cup formation [28]. However, we did not find particles in the cytoplasm at 5 or 10 min. In line with previous results [6], we found particles in the cytoplasm at 30 min (Additional file 1: Fig. S5a).

We also studied mitochondrial morphology and found mostly normal mitochondria with clear cristae or elongated mitochondria with cristae at 10 min exposure. At 60 min, significantly more mitochondria exhibited morphological changes (dissolved or dark matrix) (Additional file 1: Fig. S5b). These data indicate that the early silica-induced depolarization and DDR preceded uptake of silica particles. Instead, depolarization and DNA damage coincided with particles interacting with the plasma membrane and an active remodeling of the cell surface.

**Fig. 7:**
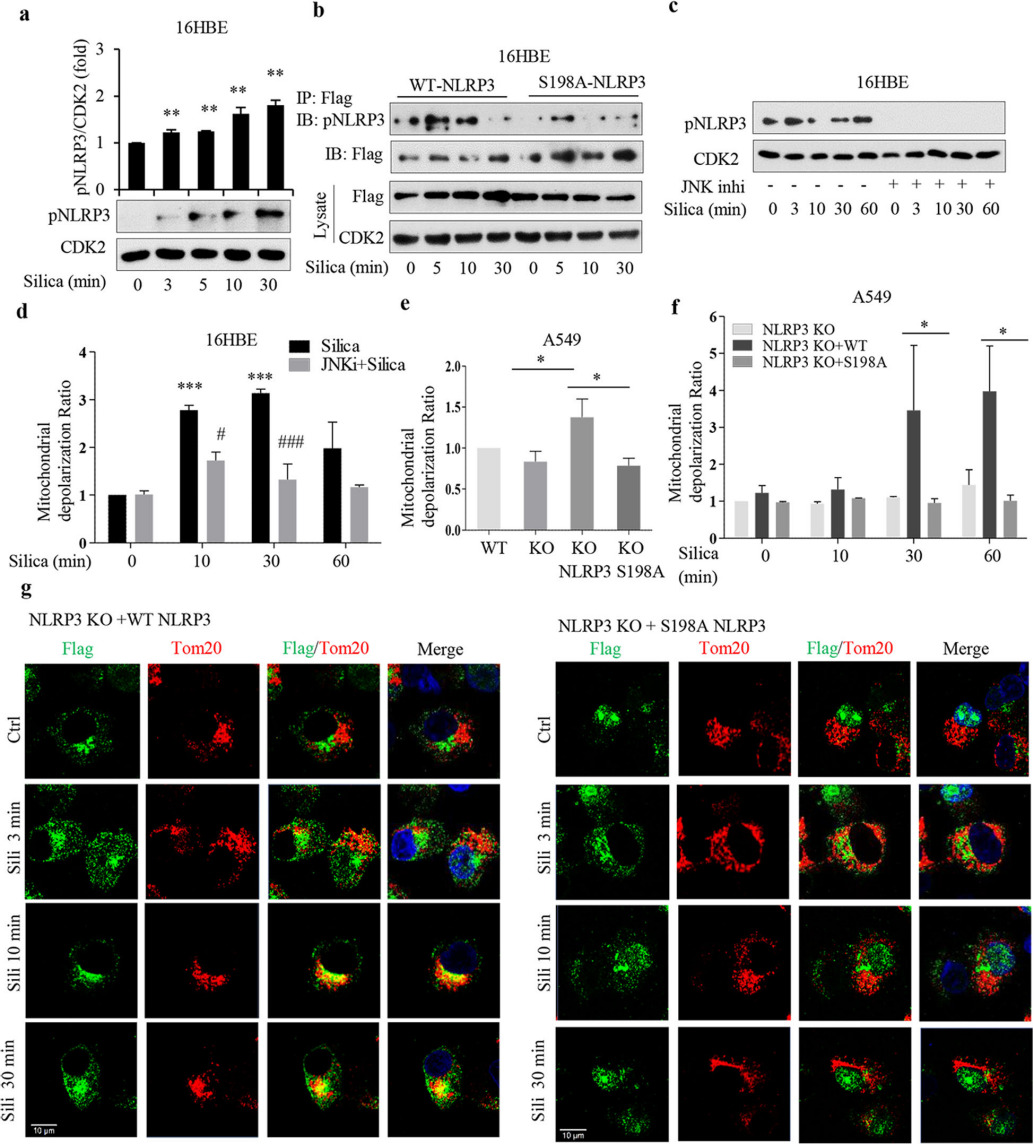
Silica-induced phosphorylation of S198 NLRP3 leads to mitochondrial depolarization. **a** Western blot analysis of S198 phosphorylated NLRP3 (pNLRP3 in 16HBE cells treated with silica (5 μg/cm^2^) for times indicated. **b** Immunoprecipitation analysis of pNLRP3 (S198) in 16HBE cells transfected with WT NLRP3-Flag or mutant S198A NLRP3-Flag for 24 h and thereafter exposed to silica for times indicated. **c** Western blot analysis of pNLRP3 (S198) in 16HBE cells pretreated (1 h) with JNK inhibitor (10 μM) and thereafter treated with silica (5 μg/cm^2^) for times indicated. **d** Mitochondrial membrane potential (JC-1 staining) in 16HBE cells pretreated with JNK inhibitor (20 μM) and thereafter treated with silica (5 μg/cm^2^) for times indicated. **e, f** Mitochondrial membrane potential (JC-1 staining) in A549 NLRP3 KO cells transfected with WT NLRP3-Flag or mutant S198A NLRP3-Flag plasmids for 24 h (**e**) and thereafter treated with silica (**f**) for times indicated. **g** Representative confocal images showing co-localization of Flag (green) and Tom20 (red) in 16HBE cells treated as in **b**. Bars show means ± SD. All experiments were performed at least in triplicate. *p < 0.05, **p < 0.01 compared to untreated cells, or #*p* < 0.05 compared to cells not exposed to inhibitor, as determined by ANOVA

A recent study shows that a JNK1-mediated NLRP3 phosphorylation at serine 198 is essential for NLRP3 priming in LPS-primed macrophages [4]. Time aspects were compatible with our data, and we hypothesized that silica activates this signaling pathway. Employing the phosphorylation S198-specific NLRP3 antibody (pNLRP3) used in [4], we found increased levels of pNLRP3 already at 3 and 5 min after silica addition (Fig. 7a). We transfected 16HBE cells with WT NLRP3 or mutant NLRP3 (Fig. 7b). The transfection efficiency is shown in Fig. S6a (Additional file 1). In IP experiments we confirmed NLRP3 phosphorylation at 5 min after silica addition (Fig. 7b). There was a clear response also after 10 min of exposure to silica. Thereafter the signal declined. In 16HBE cells transfected with mutant pNLRP3, there was a signal at 3 min, but not at later time points (Fig. 7b), suggesting a transient phosphorylation of remaining WT NLRP3 in these cells.

JNK1 thus phosphorylates S198 NLRP3 [4], and we found that the JNK1 inhibitor VIII decreased background pNLRP3 levels and blocked silica-induced pNLRP3 formation in 16HBE cells (Fig. 7c). Particles may activate JNK1 [29], even within 5 min [30], so we tested if the JNK1 inhibitor prevented depolarization. We found that it attenuated the silica-induced depolarization at 10 min and at later time points (Fig. 7d). This finding supports the TEM data (Additional file 1: Fig. S5a), indicating that silica in contact with the cell membrane is sufficient for silica-induced depolarization.

### Overexpression of NLRP3 in NLRP3 KO cells induces mitochondrial depolarization and DNA damage

We now tested the effect of NLRP3 transfection in A549 KO cells (which did not respond to silica, Fig. 3). The transfection efficiency is shown in Fig. S6b (Additional file 1). 24 h after transfection with WT NLRP3 we found a minor, although consistent, mitochondrial depolarization (Fig. 7e). Transfection with mutant NLRP3 did not induce this effect (Fig. 7e). Silica further increased the depolarization after 30 min in WT transfected cells, but not in mutant cells (Fig. 7f). We also tested whether transfected NLRP3 co-localized with TOM20 (Fig. 7g). We found no co-localization without silica, but with silica there were indications of co-localization already at 3 min and more at 10 and 30 min. Thus, we found that silica induced a similar mitochondrial co-localization in WT transfected A549 KO cells as silica did in 16HBE cells (Fig. 5a, b). In cells transfected with serine 198 mutant NLRP3 no signs of co-localization were found, without or with silica, (Fig. 7g). These data suggest that NLRP3 *per see*, without any known activating stimuli, induced a weak depolarization. They also confirm a critical role for serine 198 phosphorylation of NLRP3 in the NLRP3 recruitment to mitochondria and depolarization induced by silica.

**Fig. 8:**
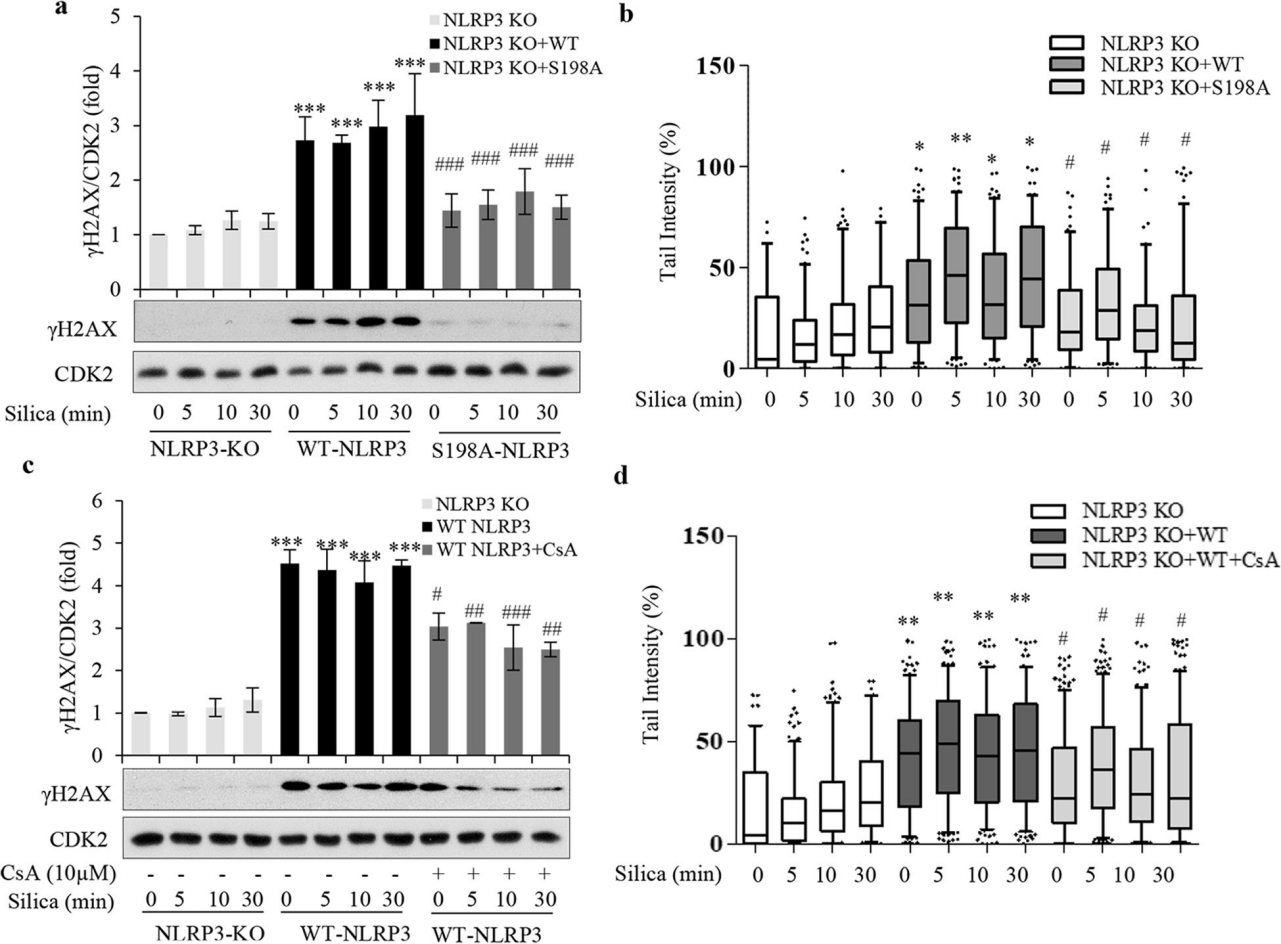
NLRP3 S198 is essential for silica-induced DNA damage. **a** Western blot analysis of γH2AX in cell lysate from A549 NLRP3 KO-cells transfected with WT NLRP3-Flag or mutant (S198A) NLRP3-Flag for 24 h and thereafter treated with silica (5 μg/cm^2^) for times indicated. **b** Comet assay analysis of DNA damage in cells from **b**. **c** Western blot analysis of γH2AX and pCHK2 in cell lysate from WT NLRP3-Flag or cyclosporine A pretreated cells (CsA, 10 μM, 1 h) and thereafter treated with silica (5 μg/cm^2^) for times indicated. **d** Comet assay analysis of DNA damage in cells from **c**. Bars in **a** and **c** show means ± SD. Boxplots in **b** and **d** show median with 95% confidence interval. All experiments were performed at least in triplicate. *p < 0.05, **p < 0.01 compared to control group, or # *p* < 0.05, ##*p* < 0.01 compared to WT NLRP3 cells not exposed to inhibitor, as determined by ANOVA

We found increased DNA damage levels 24 h after WT NLRP3 transfection in A549 KO cells (Fig. 8a-d). Transfection with mutant NLRP3 had no DNA damaging effect *per see*, and, as expected from Fig. 3d-f, silica had no effect in mutant cells (Fig. 8a, b). Furthermore, in WT transfected cells silica did not induce any significant additional increase in DNA damage levels (Fig. 8a, c) in spite an increased co-localization and depolarization (Fig. 7 f). This result clearly deviated from that shown above (Figs. 3, 4, 6). It thus seems that NLRP3 overexpression was sufficient for inducing, and saturating, the DNA damaging effect we studied.

CsA, added 23 h after WT NLRP3 transfection, gave non-significant effect on DNA damage levels (Fig. 8c, d) and as compared to the effect of CsA added prior to silica (Fig. 6e) the effect was small (Fig. 8c). This finding suggests that overexpression of WT NLRP3 induced a depolarization that peaked prior to CsA addition, and that the DDR to overexpressed NLRP3 was due to DNA damage accumulated prior to CsA addition. We thus find that transfected WT NLRP3 mimicked the silica response, albeit at a slower pace.

### Silica-induced activation of AIM2 inflammasome and non-homologous end joining (NHEJ)-repair signaling

To further investigate an early DDR induced by silica we tested if silica particles also induced AIM2, which has been shown to sense cytoplasmic DNA and DNA damage (double strand breaks, DBS) induced by IR [31]. Somewhat delayed as compared to NLRP3, we found increased AIM2 levels (Additional file 1: Fig. S7a). The delay was expected, as JNK1 did not phosphorylate AIM2 in the same context as it phosphorylated NLRP3 [4]. Instead, the early silica-induced DDR can explain the delayed AIM2 activation. In a previous 24 h mouse study silica was shown to activate STING, another sensor of damaged DNA [32].

To further confirm an early DNA damage response, we assessed DNA repair signaling in silica-exposed cells. By employing the LION tool [33] (http://lbd.lionproject.net/) we explored connections between the three factors: NLRP3, mitochondrial dysfunction and DNA repair, and the tool identified ligase IV (LIG IV) was as a possible connection. In a final step of non-homologous end joining (NHEJ), LIG IV forms complexes with XRCC4 in DNA-PK-dependent NHEJ repair of DSBs [34, 35], and we investigated co-localization of LIG IV and XRCC4 employing confocal microscopy.

**Fig. 9:**
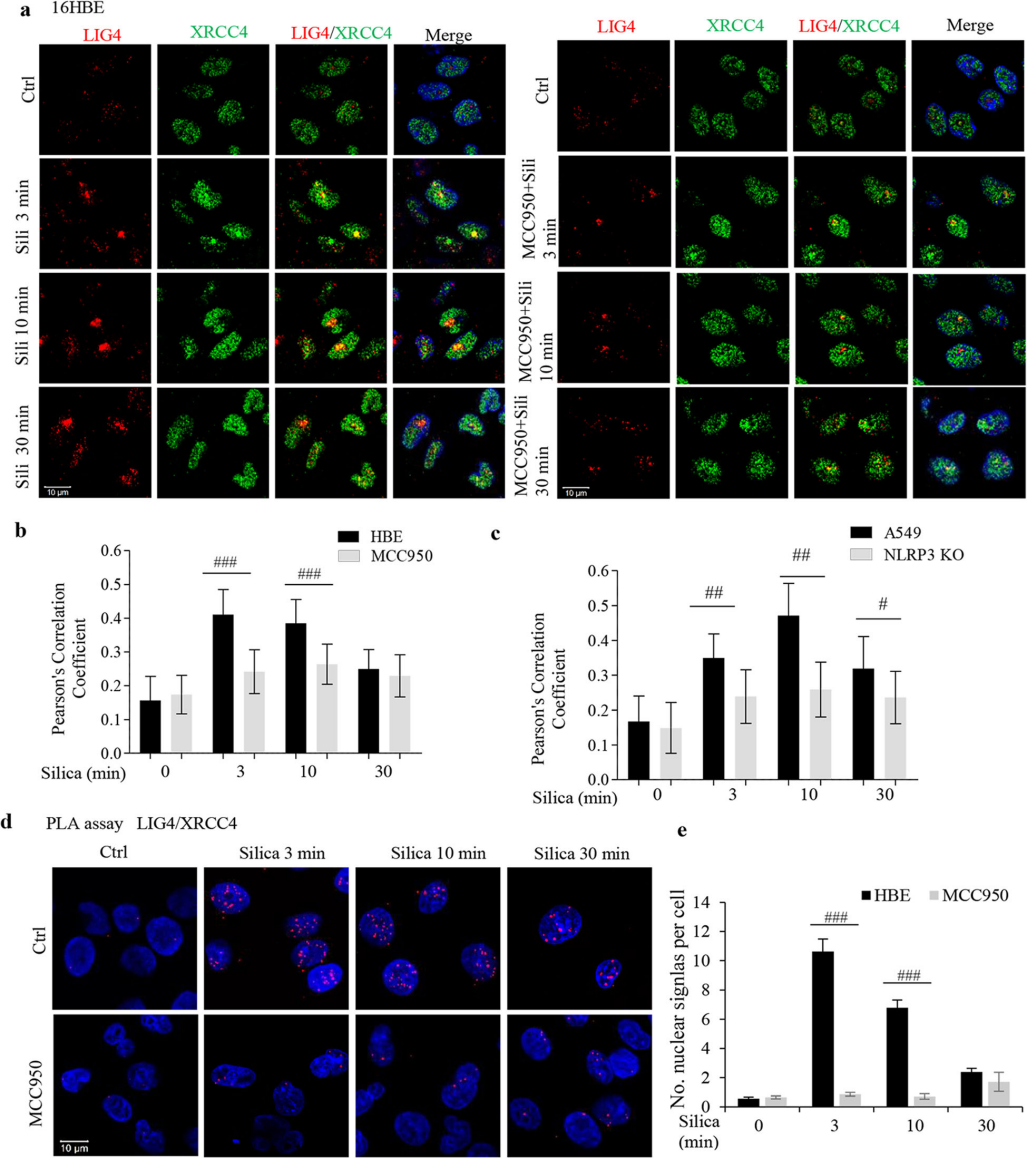
Silica rapidly induces co-localization of NHEJ repair proteins. **a** Representative confocal images showing co-localization of XRCC4 (green) and LIG 4 (red) in 16HBE cells pretreated with MCC950 (500 nM, 1 h) as indicated, and thereafter exposed to silica (5 μg/cm^2^) for times indicated. Nuclei were stained with DAPI (blue). **b** The co-localization was quantified using Pearson’s correlation coefficient. Data from 100 cells was analyzed. **c** Confocal microscopy analysis of co-localization of XRCC4 and LIG 4 in A549 WT and A549 NLRP3 KO cells treated with silica (5 μg/cm^2^). Images shown in Supplementary Fig. 7b was analyzed using Pearson’s correlation coefficient. **d** Co-localization of XRCC4 and LIG 4 was assessed by PLA assay in cells from **a**. Red dots indicate proximity for LIG4 and XRCC4. Nuclei were stained with DAPI (blue). **e** Quantification of the number of dots. All experiments were performed at least in triplicate. #*p* < 0.05, ##*p* < 0.01, ###*p* < 0.001 compared to non-transfected cells or cells not exposed to inhibitor, as determined by ANOVA

Consistent with estimates that in a dividing cell an average of ten DSBs occur per day [34], we found very few co-localizations of LIG IV and XRCC4 in 16HBE control cells (Fig. 9a). However, we found more already at 3 min after silica addition. This increase was prevented by the ASC/NLRP3 complex inhibitor, MCC950. We also compared WT A549 and NLRP3 KO A549 cells and less co-localization was seen in KO cells (Fig. 9c, Additional file 1: Fig. S7b). The PLA assay (Fig. 9d, e) showed that proximity between these two repair proteins peaked at 3 min. Within 30 min, irrespective of techniques employed but despite maintained DDR signaling (see e.g. Figure 3a, d), the repair signal returned to background levels. The NHEJ repair signaling confirms an early DNA damage response. In addition, the involvement of LIG IV and XRCC4 indicate an error prone DNA repair [35], which may cause mutations.

### Silica induces an early DNA damage response in vivo

We studied rapid effects of low dose of silica in mice. Mice express the NLRP3 inflammasome proteins in bronchiolar epithelia [36], and we exposed mice intra-nasally to a single dose of crystalline silica, as described previously [37]. The dose was 25 μg per mouse, and we did not test lower doses. The tested dose is 333 times lower than the dose used in studies of autoimmunity in mice [38] or 40 times lower than the dose used to study STING activation [32]. The alveolar surface area in mice is 82 cm^2^ [39], and assuming a distribution to alveoli an inhaled dose of 25 μg will give a dose of 0.3 μg/cm^2^ in this compartment. In rats a single inhaled dose of 233 μg, but not 50 μg, provoked increased levels of IL-18 within a week [40]. A lung burden of 200 μg/rat causes persistent inflammation in sub-chronic exposure studies and the NOEL for inflammation has been estimated to be 0.3–0.7 μg/cm^2^ [16].

**Fig. 10:**
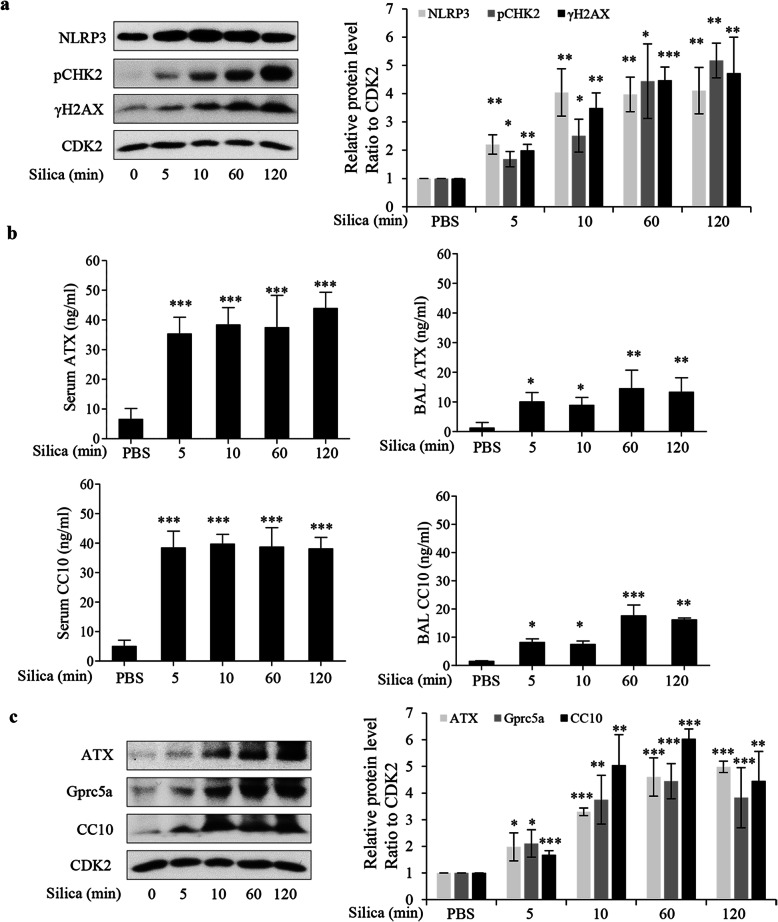
Inhaled silica particles rapidly induce DNA damage in mice. Pathogen-free mice (6 mice in each group) were exposed by intranasal instillation to silica (25 μg silica suspended in 25 μl PBS /mouse) and for times indicated or to PBS (for 10 or 120 min). **a, c** Lung tissue was homogenized in RIPA buffer. Tissue homogenate was analyzed by Western blot for NLRP3, pCHK2, γH2AX, ATX, Gprc5a, and CC10. **b** ELISA analysis of ATX and CC10 of serum and bronchoalveolar lavage (BAL) samples from mice. #*p* < 0.05, ##*p* < 0.01, ###*p* < 0.001 compared to alternatively treated cells, as determined by ANOVA

5 min after inhalation of a single dose of silica (25 μg) we found increased levels of NLRP3 and DNA damage markers (γH2AX and pCHK2) in lung homogenates (Fig. 10a) [41]. This suggested a similar early DNA damaging response in vivo as we documented in vitro.

Our previous study documented an involvement of an ATM-dependent ATX secretion in the DDR to silica, and even 0.1 μg silica/cm^2^ induced secretion in cell models [6]. We have also shown that the ATX product LPA in plasma is a sensitive biomarker for lung toxicity in humans [42, 43]. Employing 16HBE cells and 5 μg silica/cm^2^, we found extracellular ATX at 3 min (Additional file 1: Fig. S8a) as well as increased intracellular levels of NLRP3 and DNA damage markers (Additional file 1: Fig. S8b). In vivo we found increased levels of ATX in serum and in BAL already at 5 min, and these levels remained elevated for 2 h (Fig. 10b), consistent with a rapid DNA damaging effect of silica inhalation in vivo.

Furthermore, we analyzed CC10, also known as CC16. CC10 has been used as biomarker for silica effects in humans [44] and is mainly produced by epithelial club cells in distal respiratory and terminal bronchioles [45]. In mouse lung homogenate we found increased levels of CC10 at 5 min and later. These levels paralleled the ATX levels (Fig. 10c), supporting an involvement of epithelial cells in distal parts of the airway epithelium. We also found a rapid CC10 response in serum and BAL (Fig. 10 b) which is consistent with a release of ATX, extracellular formation of LPA (as described previously [6]) and subsequent CC10 release. Interestingly, the response in BAL for both CC10 and ATX was somewhat delayed as compared to that in serum, suggesting that blood was the primary target for the release of these signaling molecules.

We also analyzed GPRC5A, a G protein-coupled receptor, as a potential biomarker. GPRC5A is mainly expressed in end bronchiolar epithelium [46, 47], and Gprc5a −/− mice are sensitive to silica-induced tumorigenesis [48] and is downregulated in lung adenocarcinomas [49]. Furthermore, Gprc5a −/− mice are sensitive to acute lung effects [50] involving ATX responses [51]. In vivo, Gprc5a levels gradually increased in lung tissue (Fig. 10c), confirming an involvement of distal parts of the airway epithelium in the in vivo experiments. Taken together, the in vivo data indicate an equally rapid DDR as our in vitro data. They also implicate epithelial cells in this early response.

## Discussion

In this study we show that crystalline silica in low doses rapidly (within 5–10 min) induce DNA damage accumulation in airway epithelial cells. We were unable to correlate this early DDR to an increased ROS production but found that particles in contact with the cell membrane induce NLRP3 phosphorylation, pNLRP3-mitochondrial co-localization, and mitochondrial depolarization. These events were necessary for the early DNA damage accumulation. We saw an equally rapid DDR in vivo, and our data suggest a novel signaling pathway for inducing DNA damage that should be of relevance for silica-induced carcinogenesis.

Our data indicate a role for a NLRP3-dependent mitochondrial depolarization in silica-induced DNA damage. Different lines of evidence support this. Firstly, a similar mitochondrial depolarization and DNA damaging effect was induced by FCCP. Secondly, we showed that silencing NLRP3 by siRNA and CRISPR CAS9 prevented both mitochondrial depolarization and DNA damage. We also showed that transfection of KO NLRP3 cells with WT NLRP3 *per see* induced depolarization and DNA damage (Fig. 7e, Fig. 8). Furthermore, stabilization of the mitochondrial membrane potential prevented silica-induced DNA damage without affecting the silica-induced NLRP3 response (Fig. 6e). These data indicate that silica particles induce a mitochondrial depolarization that is critical for the rapid DDR and depends on serine198 phosphorylation of NLRP3.

Silica and FCCP co-localized NLRP3 with mitochondria. Our data indicate a role for pNLRP3 protein in this co-localization, but do not show whether NLRP3 was activated or not. In a macrophage context, serine198 phosphorylation of NLRP3 is described as a priming event [4, 52]. In our model, it is possible that a fraction of pNLRP3, perhaps without ASC (i.e. non-activated) co-localized with mitochondria, whereas another fraction activated the inflammasome and IL-1β. This interpretation is supported by the depolarization seen in WT transfected cells (Fig. 7e), and by a study of hypoxia in renal epithelial cells [53] which shows that NLRP3 without ASC interacted with and depolarized mitochondria. Another study, using HeLa cells, did not find any co-localization between NLRP3 and mitochondria [13]. However, that study [13] differs in many aspects from our study. Thus, we investigated particles, plasma membrane-triggered depolarization, and we employed pNLRP3 antibodies and bronchial cells. It also differs from [53], which used hypoxia and renal cells. By relating our data to those in [13, 53], it becomes evident that cell-origin and/or stimuli may influence NLRP3-dependent responses in epithelial cells. The respiratory epithelium is adapted to constant exposures to microorganisms and particles [54], so particle-specific responses seem plausible.

Despite their physical and chemical differences, crystalline silica and FCCP apparently triggered a common series of events leading to DNA damage. FCCP depolarizes not only the mitochondrial membrane but also the cell membrane [21], and this plasma membrane effect might explain the FCCP-induced canonical NLRP3 activation shown here as caspase1 and IL-1β activation. FCCP depolarize the plasma membrane within 10 s and earlier than the mitochondrial membrane [21]. EC_50_ for cell membrane depolarization was 410 nM [21], which fits our data and is lower than that significantly increasing ROS levels [55]. However, other NLRP3-stimulating mitochondrial toxins may act differently [56], and our data do not exclude a primary mitochondrial effect of FCCP.

We found no correlation between an increased ROS level and DNA damage accumulation, and this finding distinguishes the present study from many previous studies [6, 16, 29]. However, we do not exclude a role for ROS signaling. For example, it is possible that we did not detect subtle increases in ROS signaling that e.g. triggered the mitochondrial effects. Furthermore, we cannot exclude that ROS contributed to the DDR in an additional way. Thus both NLRP3 [22] and AIM2 [57] have been reported to inhibit DSB repair, effects that perhaps permit that even minor and undetected increments in ROS levels resulted in a detectible DNA damage response. These aspects need further studies.

A study on silica-promoted lung cancer shows that there was an exponential increase in the number of lung tumors per mouse with increasing expression IL-1β in the lungs [58]. This and other studies [59, 60] suggests a role for NLRP3 in lung carcinogenesis. A similar scenario was recently suggested for H. Pylori-induced gastric cancer. This study indicates a proliferative effect in epithelia and a carcinogenic role for upregulated epithelial NLRP3 in the development of mouse and humans gastric cancer [61]. Furthermore, AIM2 senses e.g. IR-induced DSBs within 60 min [31], and a role for AIM2 overexpression in A549 cell proliferation and epithelial mesenchymal transition (EMT) in non-small cell lung cancer was recently suggested [62]. Collectively, these studies suggest classical tumor-promotive effects of NLRP3 and AIM2 in epithelial cells. This is in line with our data implicating GPRC5A. GPRC5A confers resistance to tumor-promotive effects of silica [48]. GPRC5A also prevents acute lung injury [50] and lung cancer [63, 64] and is mainly expressed in distal, flat bronchiolar epithelium [46, 47], which also express NLRP3 [36]. This distal area has been implicated in lung carcinogenesis [65, 66] and is ascribed a specific role in silica-induced carcinogenesis [6, 15, 16].

The above discussion together with the activated DDR and NHEJ error prone DNA repair, suggest that silica particles activate both mutagenic and localized tumor-promotive effects. Our findings thus connect silica to the initiation/promotion concept for tumor development. They may for example explain the supra-linear hyperplasia (which preceded fibrosis and an accelerated accumulation of inflammatory cells) that was seen in rats inhaling 15 mg/m^3^ silica for 28 days [67]. A role for the initiation/promotion concept in human lung carcinogenesis was recently indicated by a mutation analysis of human normal lung tissue [68].

In line with previous studies [14–16], our data indicate that crystalline silica acts as a so called “threshold carcinogen”, but challenge the notion that particle uptake is necessary for cell-intrinsic epithelial DNA damaging effects [16]. The rapid in vivo DNA damage response to a low single dose (25 μg/mouse) of silica particles also argues against a dependence of airway macrophages in early epithelial DNA damage. Studies of early effects in macrophages indicate a wider time scale [69].

## Conclusions

Using airway epithelial cell models, we show that crystalline silica particles rapidly phosphorylate NLRP3 and induce a NLRP3-dependent mitochondrial depolarization. Within 5–10 min this leads to DNA damage accumulation. We saw an equally rapid DDR in mice inhaling a low dose of silica particles. After evaluation, our data might be informative for developing models for assessing exposure levels that do not cause genotoxicity.

## Methods

### Cell culture

Human Bronchial Epithelial Cell 16HBE14o- (16HBE) is immortalized human bronchial epithelial cells transformed with SV40 large T-antigen. It was provided by Prof. Dieter C. Gruenert (University of California, San Francisco, CA; Cozens). The cells were grown in plates coated with collagen and were cultured for no more than 6 passages in order to maintain the phenotype. A549 (ATCC® CCL-185™) was obtained from ATCC (Manassas, VA, USA). The 16HBE cells were maintained in EMEM (Bio Whittaker, Lonza) supplemented with 10% (v/v) inactivated fetal bovine serum (FBS) (Gibco, Fisher Scientific, USA), 100 U/ml penicillin and 100 μg/ml streptomycin mixture (Gibco, Fisher Scientific, USA) and 2 mM L-glutamine (Gibco, Fisher Scientific, USA). A549 cells were grown in DMEM (Gibco, Fisher Scientific, USA) medium supplied with 10% (v/v) FBS, 100 U/ml penicillin and 100 μg/ml streptomycin mixture and 5 mM sodium pyruvate (Gibco, Fisher Scientific, USA). All cells were cultured in a humidified atmosphere at 37 °C with 5% CO2. Used cell lines were authentizised as described previously [6].

### Antibodies and reagents

Antibodies against NLRP3 (HPA012878), Tom20 (WH0009804M1), Flag (F1804), XRCC4 (UM500021), and Gprc 5a (HPA007928) were purchased from Sigma-Aldrich (St. Louis, MO, USA). pCHK2 (Tyr68, 2661S), GAPDH (5174P) and γH2AX Ser139(2577 L) were from Cell signaling (Beverly, MA). Caspase-1 (SC-56036), IL-1β (SC-52012), ASC (SC-271054), DNA PKcs (SC-390698), LigIV (SC-271299), CC-16 (SC-365992), and CDK2 (SC-163) were from Santa Cruz (Santa Cruz, CA, USA). Mito TEMPO, carbonyl cyanide 4-(trifluoromethoxy)-phenyl-hydrazone (FCCP), Antimycin A, MCC950 and Cyclosporin A (CsA) were purchased from Sigma-Aldrich (Sigma-Aldrich, St. Louis, MO, USA). JNK inhibitor VIII was purchased from Calbiochem (San Diego, CA). All the chemical stocks were prepared according to SDS information provided by company and stored in − 20 °C. The reagents were dissolved in dimethyl sulfoxide (DMSO) with the final concentration of DMSO was < 0.1%. No effect of DMSO was observed. A specific antibody against S198 phosphorylated NLRP3 was kindly provided by Professor Tao Li from National Center of Biomedical Analysis, China.

### Silica particle preparation and characterization

Crystalline silica particles (Min-U-Sil 5) were purchased from U.S. SILICA Company. Nominal diameter was 1.6 μm. Particle dispersions were characterized to measure the size distribution by Dynamic light scattering (DLS) and the zeta potential using A Malvern Zetasizer (Malvern Nano-ZS90, UK), as described previously [6]. Silica particles were UV-irradiated overnight and the silica particles were dispersed by sonication for 15 min before addition to culture medium. In order to further verify whether particles are free of endotoxins or lipopolysaccharides (LPS), the Limulus Amebocyte Lysate (LAL) test (QCL-1000 kit, Lonza group ltd., Switzerland) was used. Result shown that the concentration of endotoxin in silica particle was 0.03 endotoxin units (EU) that was much lower than the EU regulation (0.5 EU/mL), indicating that the particles are free of endotoxin. Endotoxin contamination evaluation via LPS was detected in silica particle by the endpoint chromogenic Limulus Amebocyte Lysate (LAL) method using the QCL-1000 kit (Lonza group Ltd., Switzerland) according to the manufactory’s protocol. Briefly, silica particle was diluted in endotoxin free water with concentration of 50 μg/ml. LPS spiked samples were used as positive controls. Endotoxin standards (0.1 EU/mL to 1 EU/mL) prepared in endotoxin free water were used for standard curve calculation. The color change was measured at 540 nm by a micro-plate reader (TECAN, Zurich, Switzerland). In addition, the oxidative capacity of silica particles was analyzed by cell-free ROS generation through H2DCFDA and result shown no ROS generation.

### NLRP3 knock-out cell line by CRISPR CAS9

gNLRP3 CRISPR CAS9 plasmids were kindly provided by Dr. Isak Dermirel and Prof. Katarina Persson (Örebro Univerisity, Sweden). Guide RNA sequence: gNLRP3 1 GGCTGCATTCCCCCTCCGAG; gNLRP3 2 GCTAATGATCGACTTCAATG. The transfection method was described previously [70]. In brief, A549 cells plated in 6-well-plates (5 × 10^5^ cells per well) were transfected with different gRNA targeting NLRP3 or empty gRNA plasmids. After transfection for 48 h, single cells were treated with puromycin (Sigma-Aldrich, St. Louis, MO, USA). After 4–5 weeks, NLRP3 KO cell lines were selected and characterized by functional tests, evaluating NLRP3 knock-out efficiency and NLRP3 activation.

### Small interference RNA (siRNA)

Interference RNA (siRNA) against NLRP3 and control scramble siRNA were purchased from Santa Cruz Biotechnology (Santa Cruz, CA, USA). 16HBE cells were seeded in a 6-well-plates (2,5x 10^5^cells/well), and transfected with Lipofectamine RNAiMAX (Fisher Scientific, New Hampshire, USA) and siRNA for 72 h.

### NLRP3 plasmids and cell transfection

pCMV6-NLRP3 and pCMV6-NLRP3 S198A plasmids were kindly provided by Prof. Tao Li from National Center of Biomedical Analysis, China. Briefly, 16HBE cells or A549 NLRP3 knockout cells were seeded in a 6-well-plates (5 × 10^5^ cells/well), and transfected with the plasmids and lipofectamine 3000 (Fisher Scientific, New Hampshire, USA) for 24 h.

### Animal treatment and silica exposure

Pathogen-free male C57BL/6 mice (8–12 weeks old) were purchased from Charles River (Sulzfeld, Germany). The animals were housed in plastic cages with absorbent bedding material and were maintained on a 12 h daylight cycle. Food and water were provided ad libitum. Mice were treated as described [37]. In brief, mice were treated with a short isoflurane anesthesia and thereafter exposed to silica by intranasal instillation (25 μg suspended silica in 25 μL PBS) for 5, 10, 60 and 120 min. Control mice were challenged with PBS. Thereafter the animals were deeply anesthetized and sacrificed. Blood was collected by heart puncture in 2 mM EDTA and serum was obtained by centrifugation and stored at − 80 °C for further analysis. The mice were then tracheoctomized and bronchoalveolar lavage (BAL) was performed by two consecutive instillations of 0.4 mL of cold PBS through the tracheal cannula, followed by gentle aspiration. The recovered BAL fluid was pooled and placed on ice until its separation into cell-free BAL fluid for further analysis. Lung tissues were collected and dry frozen at − 80 °C for further analysis.

The experiments involving animals were conducted according to Swedish governmental norms. Authors have complied with all relevant ethical regulations for animal testing and research. This study was approved by ethical permit N55/15 from Stockholm ethics committee. We have complied with relevant ethical regulations.

### Homogenization of lung tissue

Lung tissues from mice were cut into pieces on ice and thereafter RIPA buffer (50 mM Tris-HCl, 150 mM NaCl_2_, 1 mM EDTA, 1% NP-40, 0.25% deoxycholic acid, pH 7.4) (Sigma-Aldrich, St. Louis, MO, USA) was added, containing protease inhibitor cocktail (Roche) and PMSF. Lung tissues were homogenized by using Tissuelyser LT (Qiagen, Hilden, Germany) at 50 Hz for 2 min. Thereafter the lysates were centrifuged (14,000 x g) at 4 °C for 10 min. The supernatants were stored at − 80 °C until use.

### Mitochondrial ROS and total cellular ROS measurement

Mitochondrial ROS production was assessed with Mito SOX red probe (Fisher Scientific, New Hampshire, USA) according to the manufactory’s protocol. The cells were seeded in a black 96-well plate. After exposure, the cells were washed with HBSS (Gibco, Fisher Scientific, New Hampshire, USA), and incubated with 5 μM Mito SOX red probe for 15 min. After washing with HBSS, the fluorescence intensity was measured at an excitation wavelength of 510 nm/emission at 590 nm by micro-plate reader (TECAN, Zurich, Switzerland).

Total cellular ROS measurement was evaluated by H2DCFDA according to the manufactory’s protocol (Fisher Scientific, New Hampshire, USA). After washing with PBS, the cells were incubated with 10 μM of DCFDA at 37 °C in dark for 30 min. After washing with HBSS, the fluorescence intensity (excitation = 485 nm; emission = 530 nm) was measured using a micro-plate reader (TECAN, Zurich, Switzerland).

### Mitochondrial membrane potential measurement

JC-1 (Fisher Scientific, New Hampshire, USA) and TMRE (Abcam, CA, USA) were employed to investigate the mitochondrial membrane potential according to the manufacturer’s protocol. JC-1 was employed to assess mitochondrial depolarization and TMRE was used to evaluate the mitochondrial function. For JC-1 staining, cell pellets were collected and re-suspended in culture medium containing 2 μM of JC-1 and incubated in the dark at 37 °C for 20 min. After washing the cells were diluted (5 × 10^5^ cells/ml) in PBS and measured by a flow cytometer (BD Accuri C6, BD Biosciences, NJ, USA) at an excitation wavelength of 488 nm/emission at 523 nm for the J-monomer form and 590 nm for the J-aggregate form. For TMRE staining, the cells were seeded in a 96-well plate (1.5 × 10^4^ cells/well) overnight. The cells were pre-incubated with 50 nM TMRE for 15 min before exposing to chemicals. After washing with PBS the fluorescent was measured by micro-plate reader (TECAN, Zurich, Switzerland) at an excitation wavelength 540 nm/emission 590 nm.

### LDH assay

LDH release assay were performed with CytoTox-ONE™ Assay from Promega (Madison, WI, USA). In brief, the released LDH was measured from supernatant. 50 μl cell medium and 50 μl of CytoTox-ONE™ was added to each well of 96-well plate. On the parallel, the total LDH was measured from cell lysates. 2 μl of cell lysate was diluted into 50 μl with lysis buffer and mixed with the 50 μl of CytoTox-ONE™ before adding to 96-well plate. After 10 min incubation the reaction was stopped by adding stop solution and the fluorescent was measured at an excitation 540 nm/emission 590 nm by micro-plate reader (TECAN, Zurich, Switzerland).

### Annexin V and Propidium iodide (PI) staining for apoptosis

Cells were grown in 6-well plate in a density of 5x10^5^cells/well. Cells were washed in PBS and resuspended in 100 μl binding buffer and then stained with FITC-conjugated Annexin V and PI (Fisher Scientific, New Hampshire, USA). After staining, cells were diluted in 500 μl binding buffer. Flow cytometry of samples was performed using by a flow cytometer (BD Accuri C6, BD Biosciences, NJ, USA). Annexin V-FITC fluorescence (FL1) was detected through a 535/30 band pass filter, whereas PI fluorescence (FL2) was detected through a 585/40 band pass filter. Data acquisition (2 × 10^4^ events per sample) was performed using the BD Accuri C6 software (BD Biosciences, NJ, USA).

### Transmission electron microscopy

Cells were collected and fixed in 0.1 M glutaraldehyde solution. Transmission electron microscopy was performed to examine phagocytosis of silica particles and the effect on cellular mitochondrial morphology. The protocol was described in previous study [6]. In brief, the cell pellets were fixed in a 0.1 M glutaraldehyde solution. The pellets were then post fixed in 2% osmium tetroxide in 0.1 M PB, pH 7.4 at 4 °C for 2 h, dehydrated in ethanol followed by acetone, and embedded in LX-112 (Ladd, Burlington, VT). Ultrathin sections (≈60–80 nm) were cut by a Leica ultracut UCT (Leica, Wien, Austria) and contrasted with uranyl acetate followed by lead citrate and examined with in Tecnai 12 Spirit Bio TWIN transmission electron microscope (Fei company, Eindhoven, The Netherlands) at 100 kV. Digital images were captured by using a Veleta camera (Olympus Soft Imaging Solutions, GmbH, Munster, Germany).

### Immunofluorescence staining and confocal microscopy

The cells were grown on coverslips (13 mm diameter, 0.16 mm thickness) in a 12-wellplate (5x10^4^cells/ml) overnight. After exposure, the cells were fixed with 4% formaldehyde in PBS and then permeabilized with 0.2% Triton X-100, blocked with 5% FBS in 2% BSA and then incubated overnight at 4 °C with primary antibodies. The antibody concentrations were: NLRP3 1:100, ASC 1:100, Tom20 1:200, Lig IV 1:100, XRCC4 1:100, and γH2AX 1:200. After washing the cells were incubated with Alexa 488 or Alexa 594 secondary antibodies (diluted 1:200) for 1 h at room temperature. The samples were mounted with Vectashield H-1000 (Vector Laboratories Inc. UK) and sealed by nail polish. Cells were imaged using a 63x oil immersion objective on a Zeiss LSM 880 META confocal laser scanner microscope (Zeiss, Göttingen, Germany).

For co-localization analysis, images of NLRP3 with ASC, NLRP3 with Tom20, or LIG IV with XRCC4 were obtained in multi-track mode. Imaris Image Analysis Software (Bitplane AG, Zurich, Switzerland) was used to quantify the frequency of co-localization of proteins. A minimum of 50 cells per exposure condition was used for quantification. Experiments were performed at least in triplicate.

### Proximity ligation assay (PLA)

The PLA was performed according to the manufacturer’s protocol using the Duolink detection kit (Olink Bioscience, Uppsala, Sweden) with PLA probes (PLUS for mouse and MINUS for rabbit). The red spots indicated proximity between two cellular bound antibodies. For quantification, the number of detected red spots where counted. At least 50 cells were counted from three individual experiments.

### Immunoprecipitation

The cells were lysed in lysis buffer (50 mM Tris, pH 7.8, 300 mM NaCl, 1% N-P40, 1 mM EDTA, 10% glycerol and EDTA-free protease inhibitor). The cell lysates were sonicated for 30 min and thereafter centrifuged at 12,000 x g at 4 °C for 30 min. The supernatants were incubated with 1 μg of anti-FLAG antibody at 4 °C for 2 h. 30 μl of Agarose (Santa Cruz, CA, USA) was added to the complex and incubated overnight at 4 °C. Immunoprecipitates were collected and washed three times with lysis buffer and thereafter dissolved in the sample buffer for SDS/PAGE. The samples were analyzed by Western blotting.

### Western blotting

Western blotting was performed as previously [6]. The proteins in supernatants were precipitated using Acetone protein precipitation protocol. Cell pellets from supernatants or cell lysates from adherent cells were lysed by lysis buffer (IPS-7), supplied with protease inhibitors (Fisher Scientific, New Hampshire, USA) and PMSF (Sigma- Aldrich, St. Louis, MO, USA), followed by addition of blue sample buffer. Protein samples were separated by 9% or 10% SDS-PAGE with 150 V for 45 min, followed by transfer to menthol-activated PVDF membrane for 65 min at 100 V. Membranes were blocked with 5% non-fat milk in TBST at room temperature for 1 h and probed with primary antibodies overnight at 4 °C. Membranes were further incubated with HRP-conjugated secondary antibodies (diluted 1:3000) diluted in 5% non-fat milk in TBST at room temperature for 1 h. Membranes were developed using Supersignal Chemiluminescent (Fisher Scientific, MA, USA) or ECL (Amersham, Little Chalfont, UK). Densitometry analysis was performed using Quantity One software (BioRad, CA, USA). Primary antibodies employed were NLRP3 1:1000, pCHK2 1:500, γH2AX 1:1000, IL-1β 1:500, caspase-1 1:500, pNLRP3 1:500, ATX 1:200, Gprc5a 1:1000, CDK2 1:4000 and GAPDH 1:1000.

### Comet assay

Comet assay was performed as previously study [6]. After exposure the cells were embedded in low-melting-point 0.75% agarose gel, followed by treatment with lysis buffer (1% Triton, 2.5 M NaCl, pH 10) on ice in dark for 1 h. Thereafter, the slides were incubated in alkaline electrophoresis buffer for 40 min, followed by alkaline electrophoresis (300 mM NAOH, 1 mM EDTA, pH > 13) for 30 min under 25 V. The slides were neutralized with 0.4 M Tris (pH 7.8) buffer, fixed with methanol, and thereafter stained by SYBR Green (diluted 1:10000 in TAE buffer). The percentage DNA with the tail was measured with software Comet Assay IV (Instem, Stone, UK) by fluoresce microscopy (Leica, Wien, Austria).

### Elisa

ENPP2 (ATX) ELISA kit was purchased from Aviscera Bioscience Inc. (Aviscera Bioscience, CA, USA). Serum and BAL samples were analyzed according to the manufacturer’s protocol. 100 μl of each serum sample or 100 μl BAL sample was incubated in pre-coated ATX antibody plates for 2 h in room temperature. Thereafter the samples were incubated with detection antibody for 2 h and followed by incubation with HRP conjugate for 1 h. After incubation with TMB substrate solution for 15 min the reaction was stopped with stop solution. The absorbance was measured at 450 nm by micro-plate reader (TECAN, Zurich, Switzerland). Mouse serum and BAL CC 10 were analyzed by Mouse Clara Cell Protein 16 (CC16) ELISA kit was purchased from MyBiosource Inc. (San Diego, CA, USA). Followed by the manufacturer’s protocol, 50 μl of each serum sample or 25 μl of BAL sample was incubated in pre-coated CC 10 antibody plate for 1 h at 37 °C. Thereafter the samples were incubated with detection reagent A for 1 h at 37 °C, and detection reagent B for 30 min at 37 °C and by Substrate solution incubation for 10 min at 37 °C. Thereafter the reaction was stopped by stop solution. The absorbance was measure at 450 nm.

### LION literature-based discovery analysis

The LION tool [33] (http://lbd.lionproject.net/) was explored to identify associations between DNA-repair, NLRP3 and mitochondrial dysfunction. Closed discovery mode with normalized pointwise mutual information (PMI) was employed.

### Statistical analysis

All experiments were performed at least in triplicate. The statistical analysis of data of mitochondrial or cellular ROS assay, mitochondrial membrane potential assay and western blot, are presented as means ± SD; statistical differences were assessed by ANOVA with Bonferroni’s post hoc test (significance rated as *p <* 0.05). For statistical analysis of the Comet assay, the data was presented as median with 95% confidence interval; statistical differences were assessed by ANOVA with Kruskal-Wallis test and Friedman test (significance rated as *p <* 0.05). Statistical analysis and graphs were performed in Graph Pad Prism (version 5.0 GraphPad software Inc., San Diego, Calif., USA).

## Supplementary information

**Additional file 1: Figure S1.** Silica-induced ROS production does not increase until 3 h. **Figure S2.** Antimycin A increases mtROS but does not induce NLRP3. **Figure S3.** Silica and FCCP decrease mitochondrial membrane potential. **Figure S4.** NLRP3 is essential for silica- and FCCP-induced DNA damage. **Figure S5.** Silica activates cell membrane and mitochondrial alterations. **Figure S6.** Transfection efficiency of WT NLRP3 and mutant NLRP3 in 16 HBE cells or A549 KO cells. **Figure S7.** Silica-induced AIM2 in 16HBE cells and NLRP3 KO prevents silica-induced co-localization of NHEJ repair proteins. **Figure S8.** Silica rapidly induces DNA damage in 16HBE cells.

## Data Availability

The datasets supporting the conclusions of this article are included within the article and its Additional file 1.
