# Supplementary material for: Crystalline silica particles cause rapid NLRP3-dependent mitochondrial depolarization and DNA damage in airway epithelial cells
Source: Part Fibre Toxicol. 2020 Aug 10;17:39. doi: 10.1186/s12989-020-00370-2 (PMC7418441; doi:10.1186/s12989-020-00370-2)
Supplement: Supplementary file 1 — Additional file 1: Figure S1. Silica-induced ROS production does not increase until 3 h. Figure S2. Antimycin A increases mtROS but does not induce NLRP3. Figure S3. Silica and FCCP decrease mitochondrial membrane potential. Figure S4. NLRP3 is essential for silica- and FCCP-induced DNA damage. Figure S5. Silica activates cell membrane and mitochondrial alterations. Figure S6. Transfection efficiency of WT NLRP3 and mutant NLRP3 in 16 HBE cells or A549 KO cells. Figure S7. Silica-induced AIM2 in 16HBE cells and NLRP3 KO prevents silica-induced co-localization of NHEJ repair proteins. Figure S8. Silica rapidly induces DNA damage in 16HBE cells. [file 12989_2020_370_MOESM1_ESM.pdf]

Supplementary Fig. 1

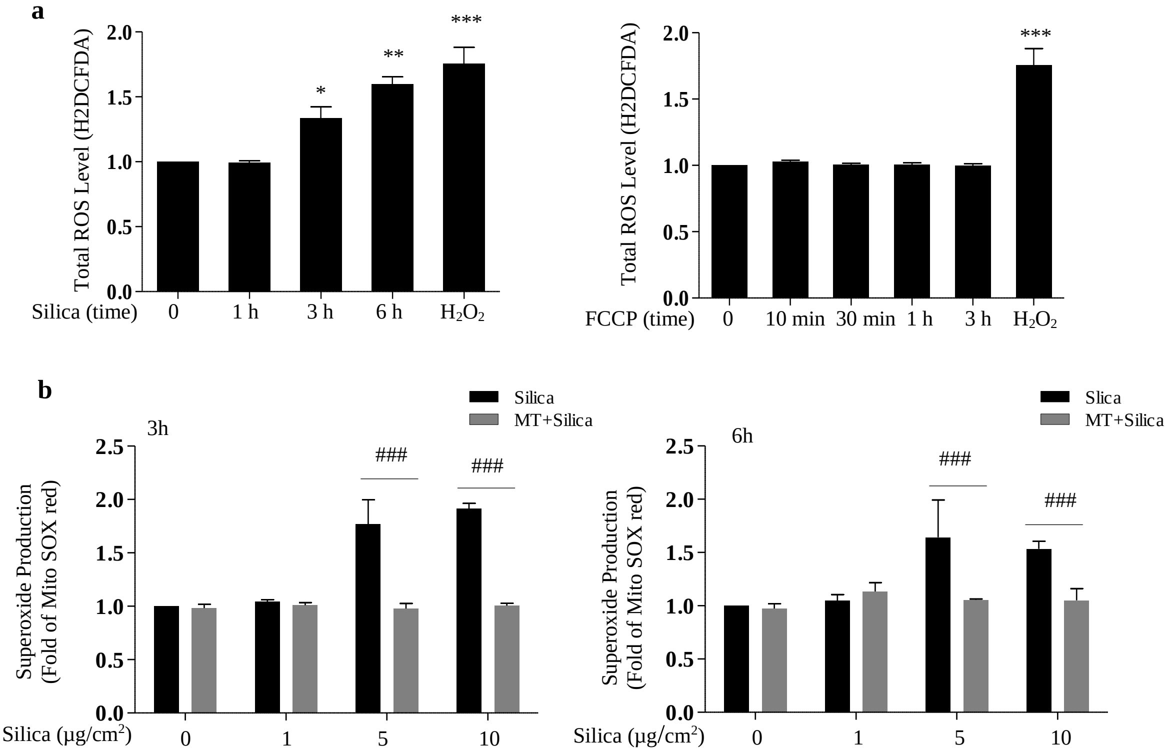

Supplementary Fig. 1 Silica-induced ROS production does not increase until 3 h in 16HBE. **a** Cellular ROS production was analyzed using H2DCFDA in cells treated with silica (5 μg/cm^2) or FCCP. H_2O_2 (1μM, 10min) was used as a positive control. **b** Mitochondrial ROS was measured in cells pre-incubated with MitoTEMPO (500 nM) for 1 h and thereafter treated with silica. Bars show means ± SD from at least three independent experiments. All experiments were performed at least in triplicate. *p < 0.05, **p < 0.01,***p < 0.001compared to untreated cells, or #p < 0.05, ##p < 0.01 compared to cells not exposed to inhibitor, as determined by ANOVA.

Supplementary Fig. 2

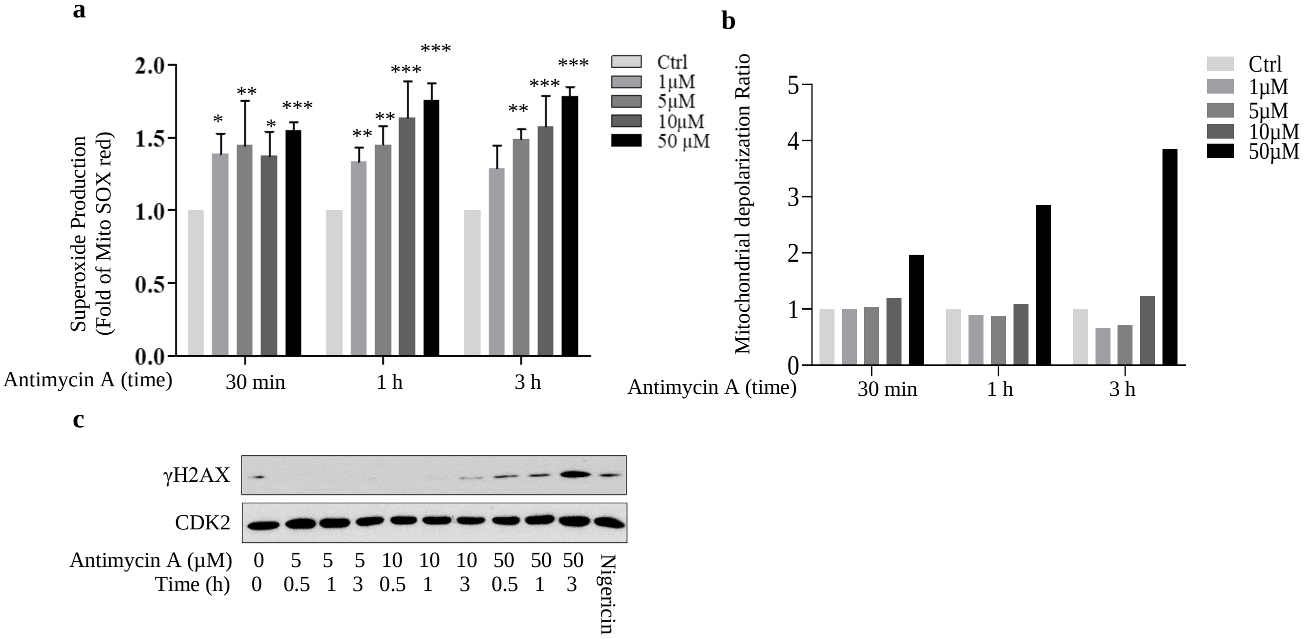

Supplementary Fig. 2 Antimycin A increases mtROS but does not induce NLRP3. **a** Mitochondrial ROS production was analyzed in 16HBE cells treated with antimycin A for times indicated. **b** Mitochondrial membrane potential was analyzed using JC-1. **c** Western blot analysis of γH2AX in cell lysates. Cells treated with Nigericin (6 µM) for 30 min was used as a positive control. CDK2 was used as loading control. Bars show means ± SD. *p < 0.05, **p < 0.01, ***p < 0.001 compared to untreated cells as determined by one-way repeated measures ANOVA.

Supplementary Fig.3

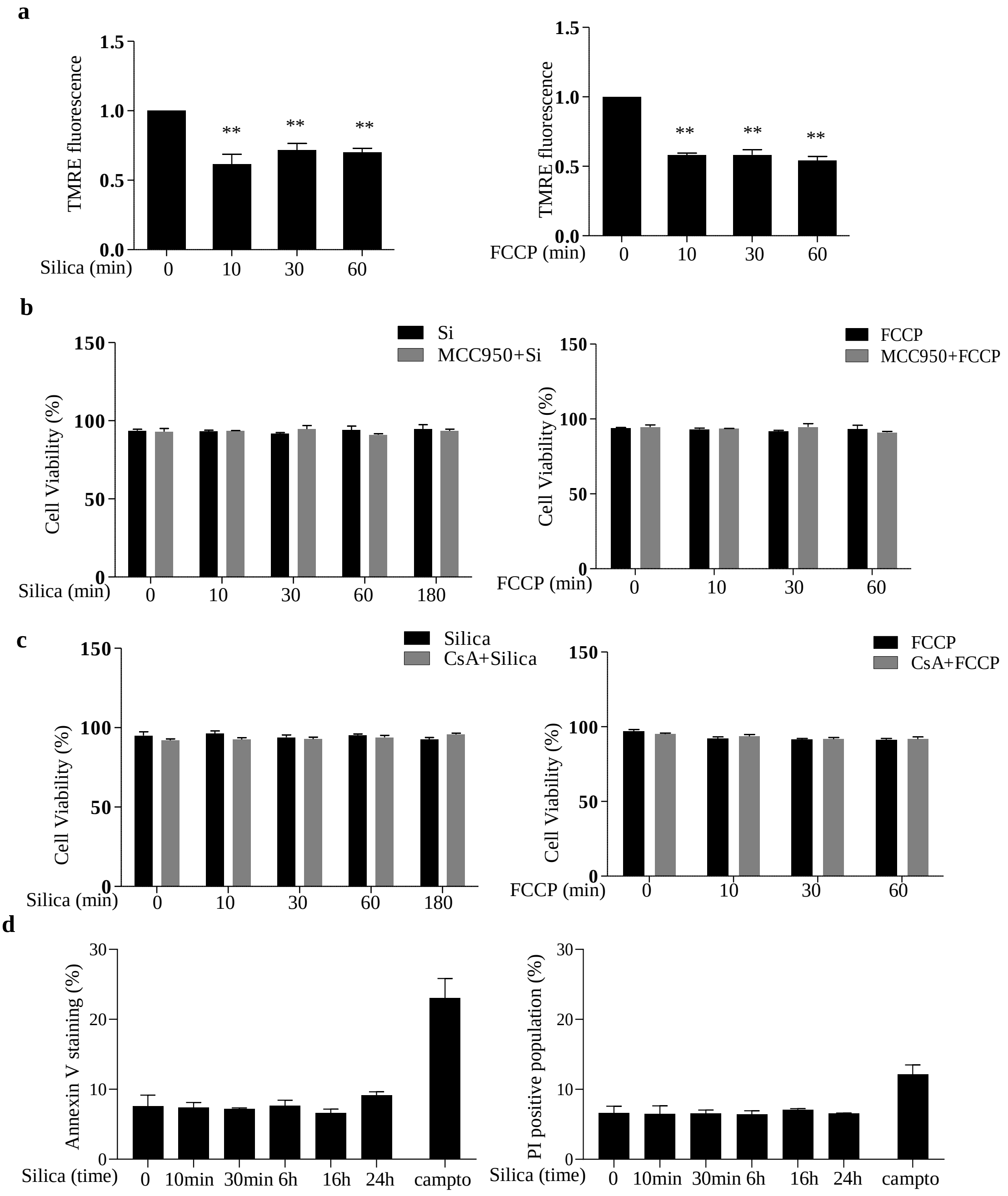

Supplementary Fig. 3 Silica and FCCP decrease mitochondrial membrane potential. **a** Mitochondrial membrane potential was analyzed using TMRE in 16HBE cells treated with silica (5 μg/cm^2) or FCCP (500 nM). **b, c** LDH assay was measured for cell viability in 16HBE cells pretreated with MCC950 (100 nM) (**b**) or with cyclosporine A (10 μM) (**c**) as indicated and thereafter treated with silica (5 μg/cm^2) or FCCP (500 nM). **d** Apoptosis assay by Annexin V and PI staining in 16HBE cells that treated with silica (5 μg/cm^2) from 10 min to 24 hrs. Treatment of Camptothecin (10uM) for 12hrs was used as positive control. Bars show means ± SD from at least three independent experiments. **p < 0.01 compared to untreated cells as determined by ANOVA.

Supplementary Fig. 4

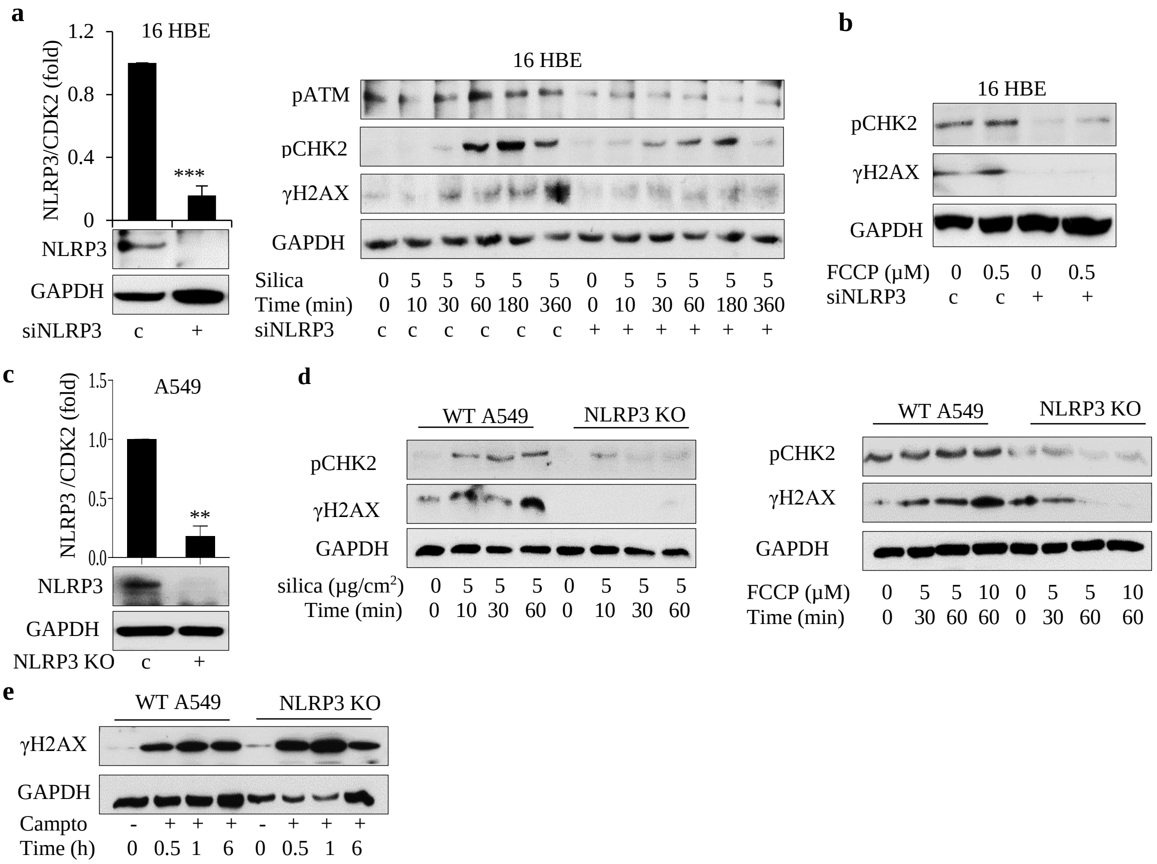

Supplementary Fig. 4 NLRP3 is essential for silica- and FCCP-induced DNA damage. **a, b** Western blot analysis of NLRP3, pATM, pCHK2, and γH2AX in cell lysates from 16HBE cells transfected with siRNA NLRP3 (+) or control siRNA (c) for 72 h and thereafter treated with silica (5 μg/cm^2) (**a**) or FCCP (500 nM, 30 min) (**b**). **c** Western blot analysis of NLRP3 in cell lysates from A549 cells transfected with gNLRP3 CRISPER CAS9 plasmids**. d** Western blot analysis of pCHK2, and γH2AX in cell lysates from A549 WT or NLRP3- KO cells treated with silica (5 μg/cm^2) or FCCP (500 nM). **e** Western blot analysis of γH2AX and pCHK2 in cell lysates from A549 WT or NLRP3-KO cells treated with Camptothecin (10 μM) for time indicated.

Supplementary Fig. 5

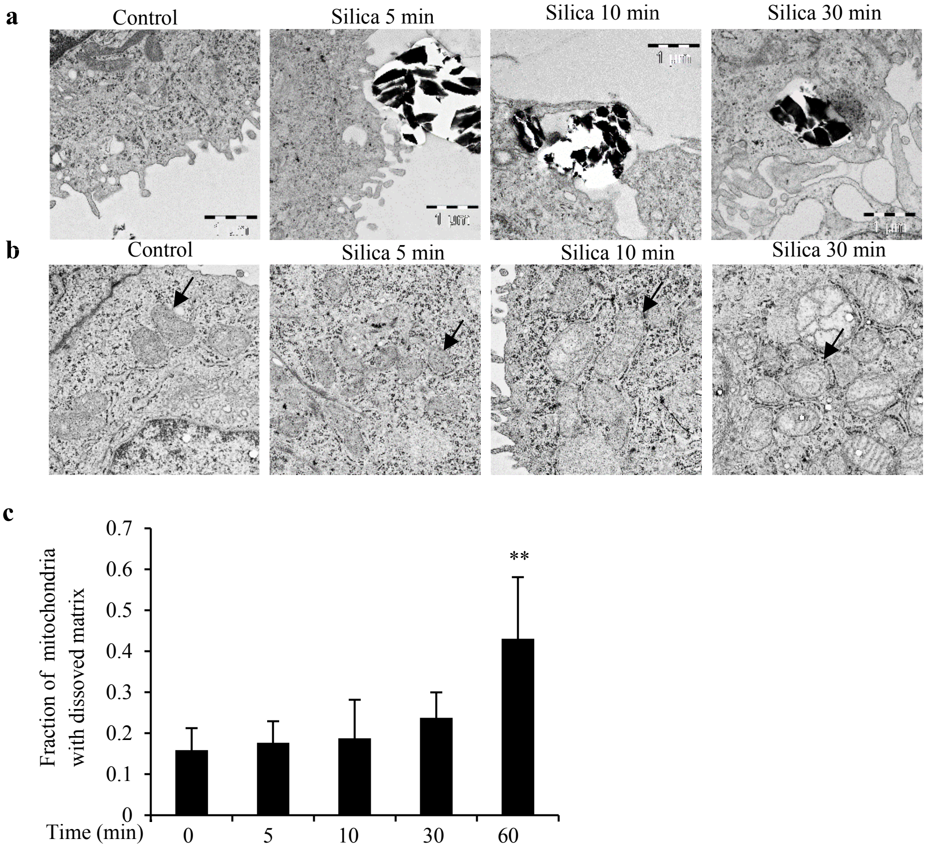

Supplementary Fig. 5 Silica activates cell membrane and mitochondrial alterations. **a** Transmission electron microscopy showed particle contact and particle uptake in 16HBE cells exposed to silica (10 μg/cm^2) for times indicated. **b** Mitochondrial morphological changes are indicated with the black arrows. **c** Bars show the fraction of mitochondria with dissolved or dark matrix. 200 mitochondria were analyzed at each time point. **$p < 0.01$ compared to untreated cells, as determined by ANOVA.

Supplementary Fig. 6

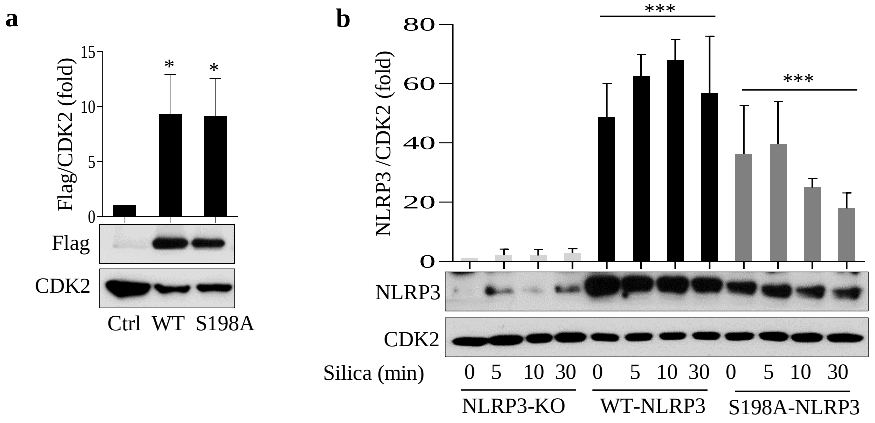

Supplementary Fig. 6 Transfection efficiency of WT NLRP3 and mutant NLRP3 in 16 HBE cells or A549 KO cells. **a** Western blot analysis of Flag in cell lysate from 16HBE cells transfected with WT Flag- NLRP3 or S198A Flag- NLRP3 plasmids for 24 hours. **b** Western blot analysis of NLRPJ3 in cell lysate from A549 KO cells transfected with WT Flag-NLRP3 or S198A Flag-NLRP3 plasmids for 24 hours. All experiments were performed at least in triplicate. *$p < 0.05$, ***$p < 0.001$ compared to control group, as determined by ANOVA.

Supplementary Fig. 7

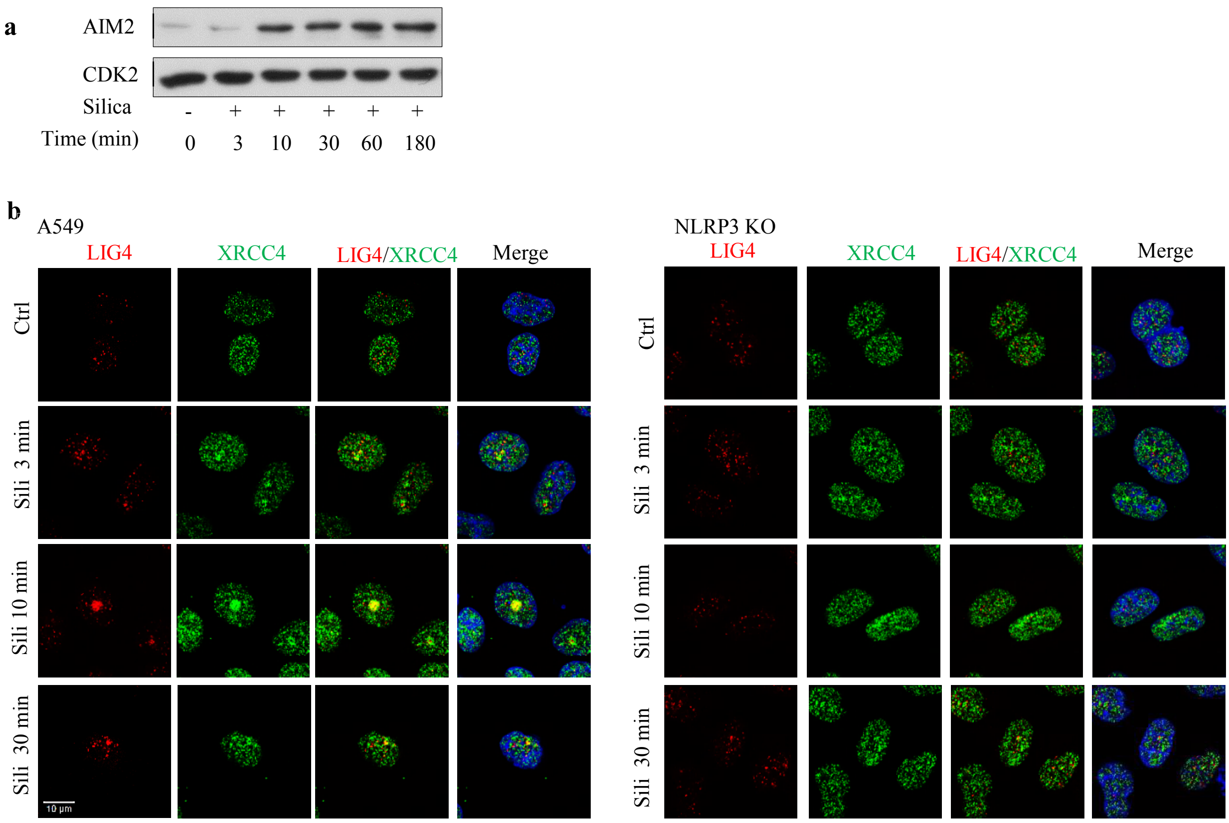

Supplementary Fig. 7 Silica induced AIM2 in 16HBE cells and NLRP3 KO prevents silica-induced co-localization of NHEJ repair proteins. **a** Western blot analysis of AIM2 in cell lysate from 16HBE cells treated with silica (5 µg/cm^2) for times indicated. **b** Confocal microscopy analysis of co-localization of XRCC4 and LIG4 in A549 WT and 549 NLRP3 KO cells treated with silica. Quantification of data is shown in Fig. 9**c**. Scale bar is 10 µm.

Supplementary Fig. 8

a

16HBE

ATX extra

H

CDK2 intra

Silica (min) 0 3 5 10 30

b

16HBE

NLRP3

pCHK2

γH2AX

CDK2

Silica (min) 0 5 10 30

Supplementary Fig. 8 Silica rapidly induces DNA damage in 16HBE cells. **a**, **b** Western blot analysis of indicated proteins in supernatants (**a**) and cell lysates (**b**) from 16HBE cells exposed to silica (5 µg/cm^2) for times indicated.
